# Amniotic fluid stem cell‐derived extracellular vesicles educate type 2 conventional dendritic cells to rescue autoimmune disorders in a multiple sclerosis mouse model

**DOI:** 10.1002/jev2.12446

**Published:** 2024-06-06

**Authors:** Giorgia Manni, Marco Gargaro, Doriana Ricciuti, Simona Fontana, Eleonora Padiglioni, Marco Cipolloni, Tommaso Mazza, Jessica Rosati, Alessandra di Veroli, Giulia Mencarelli, Benedetta Pieroni, Estevão Carlos Silva Barcelos, Giulia Scalisi, Francesco Sarnari, Alessandro di Michele, Luisa Pascucci, Francesca de Franco, Teresa Zelante, Cinzia Antognelli, Gabriele Cruciani, Vincenzo Nicola Talesa, Rita Romani, Francesca Fallarino

**Affiliations:** ^1^ Department of Medicine and Surgery University of Perugia Perugia Italy; ^2^ Extracellular Vesicles network (EV‐net) of the University of Perugia Perugia Italy; ^3^ Department of Pharmaceutical Science University of Perugia Perugia Italy; ^4^ Department of Biomedicine, Neurosciences and advanced Diagnostics (Bi.N.D) School of Medicine University of Palermo Palermo Italy; ^5^ TES Pharma Srl, I Corciano Perugia Italy; ^6^ Bioinformatics unit, Fondazione IRCCS Casa Sollievo della Sofferenza San Giovanni Rotondo Italy; ^7^ Cellular Reprogramming Unit, Fondazione IRCCS Casa Sollievo della Sofferenza San Giovanni Rotondo Italy; ^8^ Department of Chemistry, Biology and Biotechnology University of Perugia Perugia Italy; ^9^ Department of Physics and Geology University of Perugia Perugia Italy; ^10^ Department of Veterinary Medicine University of Perugia Perugia Italy

**Keywords:** amniotic fluid stem cells, autoimmune diseases, conventional dendritic cell type 2 (cDC2), dendritic cells, experimental autoimmune encephalomyelitis (EAE), extracellular vesicles, tolerogenic phenotype

## Abstract

Dendritic cells (DCs) are essential orchestrators of immune responses and represent potential targets for immunomodulation in autoimmune diseases. Human amniotic fluid secretome is abundant in immunoregulatory factors, with extracellular vesicles (EVs) being a significant component. However, the impact of these EVs on dendritic cells subsets remain unexplored. In this study, we investigated the interaction between highly purified dendritic cell subsets and EVs derived from amniotic fluid stem cell lines (HAFSC‐EVs). Our results suggest that HAFSC‐EVs are preferentially taken up by conventional dendritic cell type 2 (cDC2) through CD29 receptor‐mediated internalization, resulting in a tolerogenic DC phenotype characterized by reduced expression and production of pro‐inflammatory mediators. Furthermore, treatment of cDC2 cells with HAFSC‐EVs in coculture systems resulted in a higher proportion of T cells expressing the regulatory T cell marker Foxp3 compared to vehicle‐treated control cells. Moreover, transfer of HAFSC‐EV‐treated cDC2s into an EAE mouse model resulted in the suppression of autoimmune responses and clinical improvement. These results suggest that HAFSC‐EVs may serve as a promising tool for reprogramming inflammatory cDC2s towards a tolerogenic phenotype and for controlling autoimmune responses in the central nervous system, representing a potential platform for the study of the effects of EVs in DC subsets.

## 1 INTRODUCTION

Biological fluids normally contain extracellular vesicles (EVs), that are released by different cell types and are able to mimic specific cellular functions. These membrane particles, secreted by both eukaryotic and prokaryotic cells, play an important role in intercellular communication (Lo Cicero et al., 2015; Shao et al., 2018) by transporting a molecular cargo originating from the cell of origin (Théry et al., 2018). In 2015, a new type of amniotic fluid stem cells was identified that release EVs with remarkable immunoregulatory and tolerogenic properties in vitro and in vivo (Romani et al., 2015).

Amniotic fluid, a dynamic environment evolving during pregnancy, fulfils various functions, from nourishing the foetus to promoting growth and protecting against infection (Theis et al., 2019; Tong et al., 2009). In particular, human amniotic fluid harbours heterogeneous stem cell populations with significant therapeutic and regenerative potential (De Coppi et al., 2007). Human amniotic fluid stem cells (HAFSCs) represent a promising avenue for therapeutic exploration, unleashing EVs as a remarkable alternative to stem cell therapy (Balbi et al., 2017). Despite these advances, the exact cellular targets of these immunoregulatory EVs responsible for triggering immune regulatory functions are not yet known.

Dendritic cells (DCs), as key players of the innate immune system, possess specialized antigen‐presenting capabilities with the ability to activate naïve T cells and significantly influencing immune responses (Guilliams & van de Laar, 2015). Their heterogeneous nature includes specific characteristics in terms of origin, location, function and migration pathways (Anderson et al., 2021). Both human and murine DCs consist of major populations, namely, conventional DCs (cDCs) and plasmacytoid DCs (pDCs), which arise from bone marrow progenitor cells and differentiate into subsets that are found in different tissues (Durai & Murphy, 2016; Guilliams et al., 2016). The cDC population comprises the cDC1 and cDC2 subsets, each characterized by specific transcription factors and surface markers. The cDC1 subset, which requires the transcription factors Irf8, Id2 and Batf3, expresses characteristic markers such as XCR1, BTLA, CD8α and DEC‐205 (Durai et al., 2019). In contrast, the development of cDC2 is regulated by the transcription factors Irf4 and Notch2, and these cells are characterized by the expression of CD172a (SIRPα) as well as DCIR2 and CD11b on the cell surface (Iberg et al., 2017). Activated DCs express MHC molecules, costimulatory receptors and proinflammatory cytokines and induce T cell proliferation (Liu et al., 2021). In addition to their immunogenic functions, DCs also play a crucial role in triggering immune tolerance. They contribute to central tolerance by controlling the thymic deletion of self‐reactive T cells and the production of thymus‐derived regulatory T cells (tTreg) (Klein et al., 2019). Tolerogenic DCs (tolDCs) are the key to maintaining immunological tolerance (Yoo & Ha, 2016). They are characterized by low levels of costimulatory molecules, high levels of anti‐inflammatory cytokines (Thomson & Robbins, 2008) and expression of immunoinhibitory checkpoint targets (Barroso et al., 2021; Mohammadi et al., 2023). Recent research has shown that mature DCs enriched with immunoregulatory molecules (mregDCs) are a key element to suppress immune responses in specific microenvironments (Maier et al., 2020). Given the susceptibility of DCs to environmental modulation, it is plausible to hypothesize that EVs containing biologically active molecules, released by specific cell types, could significantly influence the plasticity and function of DCs, especially cDCs which are often dysregulated in autoimmune diseases as shown in mice with experimental autoimmune encephalomyelitis (EAE) a model of human multiple sclerosis (Heink et al., 2017; Mundt et al., 2019).

Multiple sclerosis (MS) is one of most common inflammatory and demyelinating disease of the central nervous system (CNS) (Jakimovski et al., 2023). The interplay of complex genetic and environmental factors influences multiple sclerosis susceptibility (Bjornevik et al., 2022). Despite recent advances in preventing relapses with systemic therapies, their limited efficacy in controlling the ongoing, independent progression of the disease is a major challenge. Understanding the underlying mechanisms and developing therapies that halt or prevent disease progression are key challenges in the field of MS.

The aim of the present study was to investigate whether highly characterized HAFSC‐derived EVs (HAFSC‐EVs) might be able to convert murine cDC subsets into functional regulatory cDCs and to evaluate their therapeutic role in a mouse model of central nervous system autoimmunity. Notably, we found that HAFSC‐EVs were preferentially internalized by cDC2, which are often dysregulated and highly inflammatory in autoimmune disorders, including multiple sclerosis (MS). This preferential uptake leads to the reprogramming of cDC2 towards a tolerogenic phenotype, suggesting that HAFSC‐EVs could be a potential platform for the development of regulatory DC‐based tools for the treatment of autoimmune or inflammatory diseases.

## 2 METHODS

### 2.1 Mice

C57BL/6 mice were obtained from Charles River Breeding Laboratories. Female mice, 8‐10‐week‐old, were used in in vitro and in vivo studies. All in vivo studies were in compliance with National (Italian Parliament DL 26/2014) and* Perugia University Animal Care and Use Committee* guidelines. OT‐II mice (C57BL/6 Tg(TcraTcrb)425Cbn/Crl, Crl: 643) were used for in vitro experiments. All animal studies were approved by the Bioethics Committee of the University of Perugia.

### 2.2 Cell lines

Human amniotic fluid stem cells (HAFSCs) were cultured according to Romani et al. (2015). The Mutu DC lines (DC1 and DC2) were kindly lent by Hans Acha‐Orbea (Koga et al., 2021) and cultured in IMDM+GlutaMAX (Gibco; Thermo Fisher Scientific, Inc.), with 5% FBS (ByProductos), sodium bicarbonate and antibiotics under a humidified atmosphere with 5% CO_2_ and 95% air at 37°C.

### 2.3 in vitro bone marrow‐derived dendritic cells

Bone marrow cells were isolated from C57BL/6 mice. BM was harvested from femur, tibia and pelvis using mortar and pestle in 1x PBS supplemented with 0.5% BSA and 2 mM EDTA (MACS buffer), passed through a 70 μm cell strainer and centrifuged at 1400 rpm for 5 min. Red blood cells were lysed with ACK lysis buffer (Ammonium Chloride 0.15 M, Potassium Carbonate 10 mM) and debris were removed by a gradient centrifugation using Histopaque1119 (#11191, Sigma–Aldrich) prior to culture. Cells were resuspend at 2 × 10^6^ cells/mL in Iscove's Modified Dulbecco's Media (IMDM, #12440053, Thermo Fisher) supplemented with 0.1 Non‐essential Amminoacids (#11140‐035 Thermo Fisher), 1 mM Sodium Pyruvate (#11360‐070, Thermo Fisher), 5 mM glutamine (#25030‐024, Thermo Fisher), 50 μM 2‐Mercaptoethanol (#31350‐010, Thermo Fisher), 100 U/mL penicillin, 100 g/mL streptomycin (#15140‐122, Thermo Fisher) and 10% FBS (#10270‐106, Thermo Fisher) (complete IMDM) containing 5% murine Flt3‐L and were seeded 85 mL/well in 6‐well tissue culture plates at 37°C for 8–10 days. cDC2 were purified from dendritic cells culture by magnetic sorted column (LD columns, Milteny Biotec #130‐042‐901) after 30 min incubation with a mix of biotin conjugated antibodies plus Streptavidin Negative Selection Bead (#MSNB‐6002, Thermo Fisher) or Streptavidin Positive Selection Bead (MojoSortStreptavidin Nanobeads, BioLegend). First step consisting in depletion of B220^+^ (#103204 BioLegend), CD3^+^ (#100244 BioLegend) and CD19^+^ (#115504 BioLegend) cells from total bone marrow cell culture. The second step consisting into positive selection of cDC2 cells by using biotin conjugated antibodies for CD172 (#13‐172‐182 eBioscience). First and second steps are separated by a washing step in which cells were suspended in MACS buffer and centrifuged for 5 min at 300*×*g. Purity of cDC2 preparation was evaluated by LSRFortessa (BD BioSciences) flow cytometer as B220^–^CD11c^+^MHCII^+^CD24^–^CD172α^+^. Antibodies used are: B220 PE (#103208 Biolegend), CD11c eFluor780 (#47‐0114‐82, Invitrogen), MHCII BV421 (#107632 Biolegend), CD24 PECy7 (#101822 Biolegend), CD172 PerCP‐eFluor710 (#46‐1721‐82 Invitrogen). cDC2 with a purity of >90% were used for stimulation with HAFSC‐EVs.

### 2.4 Preparation of CD4^+^ T cells

Mouse CD4^+^ T cells were prepared from spleen of wild type or OT.II mice. Total splenocytes were depleted of B220^+^ (#103204 BioLegend), CD11b^+^(#101204 BioLegend), CD11c^+^ (#117304 BioLegend) and CD8^+^ (#100704 BioLegend) cells using biotinylated antibodies for 30 min, followed by streptavidin microbeads for 30 min, and magnetic column (LD column Miltenyi Biotec) for negative separation. The negative cell fraction was suspended in MACS buffer and centrifuged for 5 min at 300*×*g and subsequently labelled with microbeads anti‐CD4 (#130‐117‐043, Miltenyi Biotec) for 30 min. After incubation, labelled cells were passed through a magnetic separation column for positive selection (LS column Miltenyi Biotec). CD4 positive fraction was eluted from column and the purity of CD4^+^ T cells was evaluated by LSRFortessa (BD BioSciences) flow cytometer as B220^−^ CD11c^−^ CD3^+^ CD8^−^ CD4^+^. Antibodies used are: B220 PeCy7 (#103222 Biolegend), CD11c eFluor780 (#47‐0114‐82, Invitrogen), CD3 APC (#100312 Biolegend), CD4 PE (#100408 Biolegend), CD8 PerCP‐Cy5.5 (#551162 BD Biosciences)

### 2.5 Extracellular vesicles isolation and cell treatment

HAFSC‐EVs were isolated from 10 × 10^6^ human amniotic fluid stem cells (at passage 6), obtained as described previously (Romani et al., 2015) according to MISEV guidelines. Specifically, amniotic fluid stem cells were cultured 24 h without serum and EVs were isolated from cell culture supernatant by differential ultracentrifugation in sterile tubes. Firstly, conditioned medium (30 mL) was centrifuge at 300*×*g for 10 min to dead cells. Supernatant was centrifuged at 2000×g for 20 min at 4°C to eliminate cell debris. The supernatant, transferred into new tubes, was centrifuged in a 45Ti rotor (Beckman) for 40 min at 10,000 × g to obtain a 10K pellet (EVs p10), and finally for 90 min at 100,000 × g to obtain a 100K pellet (EVs p100). EVs p100 pellet were washed in 50–60 mL of sterile and filtered PBS and centrifuged again at the same speed before being resuspended in 50–100 μL of sterile and filtered PBS and used for all the experiments. EVs p100 fraction are named HAFSC‐EVs in the text. To study the effect of HAFSC‐EVs on cDCs, we first performed titration of EVs in number ranging from 0.65 × 10^9^ to 5.2 × 10^9^. For the uptake studies, 2.6 × 10^9^ (i.e., HAFSC‐EVs^high^) or 1.3 × 10^9^ (i.e., HAFSC‐EVs^low^) were used for treatment of 1 × 10^6^ cells based on the previous titration results. The lowest dose of EVs (1.3 × 10^9^) was used for all functional experiments. This dosage was chosen to ensure optimal biological effects and to exclude non‐specific effects.

### 2.6 Scanning electron microscopy (SEM)

For scanning electron microscopy (SEM) analysis, samples were prepared by fixing HAFSC‐EVs p100 in 1,5% glutaraldehyde for 15 min at room temperature. A 5μl sample aliquot was deposited on glass coverslips (circular coverslips of 12 mm diameter and thickness n.1) and dried at room temperature for 15 min. Images were recorded by using a field emission gun electron scanning microscope (LEO 1525 Zeiss; Thornwood, NY, USA) with Cr metallization with a high‐resolution sputter 150T ES‐Quorum apparatus (24 s, sputter at a current of 240 mA). Chromium thickness was ∼10 nm.

### 2.7 Nanoparticle tracking analysis (NTA)

An aliquot of HAFSC‐EVs obtained as previously described were loaded into a NS500 NanoSight instrument (Malvern, UK, NTA 3.4 Build 3.4.003 version) to measure the concentration and size of the particles present in the supernatant of amniotic fluid stem cells. Constant flow injection was used, and five videos of 60 s were captured each sample by sCMOS camera and Blue488 laser type. HAFSC‐EVs p100 hydrodynamic diameter were calculated by Stokes‐Einstein equation and analysis was performed by NTA 2.3 software. Vesicles number is expressed as means ± SD. All parameters for the measurement comply with the instrument guidelines. To studying HAFSC‐EVs uptake we performed a titration in EV numbers (from 0.65 to 5.2 × 10^9^). For uptake experiments were used 2.6 × 10^9^ (HAFSC‐EVs^high^) or 1.3 × 10^9^ (HAFSC‐EVs^low^) for 1 × 10^6^ cells. For all functional experiments were used the lowest doses of EVs (1.3 × 10^9^). This dosage has been chosen to ensure optimal biological effects.

### 2.8 Western blotting analysis

Western blot analysis was performed according to standard procedures (Romani et al., 2018) in total extracellular vesicles extracts. Collected vesicles were lysed in lysis buffer (50 mM Tris HCl pH7.6 + 150 mM NaCl, 1% NP‐40, 1% SDS and a cocktail of protease inhibitors) 30 min on ice and centrifuged at 13,000*×*g for 30 min at 4°C. Proteins were resolved on SDS‐PAGE and blotted on a nitrocellulose membrane. Several antibodies were used to detect extracellular vesicles standard markers: Alix (sc‐53540) 95 kDa, CD81 (sc‐166029) 22 kDa, Tsg101 (sc‐7964) 44 kDa, ARF6 (sc‐7971) 26 kDa (Santa Cruz Biotechnology), β‐Actin (A3853) 42 kDa, and LaminA (sigma‐Aldrich) 70 kDa. Primary antibodies were diluted according to manufacturer's protocol and incubated overnight at 4°C. Secondary horseradish peroxidase‐linked antibodies were purchased from Thermo Fisher Scientific Laboratories and used as follows: anti‐mouse (#31430, 1:10000 v/v), anti‐rabbit (#31460, 1:10000 v/v) for 1 h at room temperature Membranes were developed using Clarity Western ECL Blotting Substrates (#170‐5061, BioRad) and Clarity Max Western ECL Substrate (#170‐5062, BioRad).

### 2.9 Proteomic analyses: In‐solution protein digestion and DDA

Chemicals used for protein extraction and digestion were of analytical grade, and Milli‐Q water was employed in all buffers and solutions. The HAFSC‐EVs were subjected to in‐solution digestion using 50% 2,2,2‐trifluoroethanol (TFE) in PBS and obtained peptides were desalted by solid phase extraction using the Thermo Scientific Pierce C18 Spin Columns (Thermo Fisher Scientific) (Schillaci O Sci Rep. 2017 Jul 5;7(1):4711. https://doi.org/10.1038/s41598‐017‐05002‐y). Tryptic peptides were analysed via reverse‐phase high‐pressure liquid chromatography electrospray ionization tandem mass spectrometry (RP‐HPLC‐ESI‐MS/MS) using a TripleTOF 5600 Plus System (AB SCIEX; Framingham, US) equipped with an Eksigent Nanoflow binary gradient HPLC system (nanoLC Eksigent 425 system; AB SCIEX; Framingham, US). RP‐HPLC was performed with a trap and elution configuration using a C18 reverse‐phase trap column (Acclaim PepMap 100 C18 LC Trap Column, Thermo Fisher Scientific) and the Acclaim™ PepMap™ RSLC (75 μm x 25 cm nanoViper C18 2 μm 100Å, Thermo Fisher Scientific). The reverse‐phase LC solvents were solvent A (0,1% FA in water) and solvent B (0,1% FA in 98% acetonitrile). Three technical replicates (4 μg each) were acquired in Data Dependent Acquisition (DDA) mode. Each replicate was loaded in the trap column at a flow rate of 5 μL/min for 10 min employing a solvent, from loading pump, containing 2% acetonitrile + 0.1% v/v TFA in water and eluted and was eluted at a flow rate of 300 nL/min using a gradient method according to which solvent B is linearly increased from 2% to 30% within 120 min and then to 60% within 15 min; afterwards, phase B is further increased to 95% within 2 min. Then, phase B is maintained at 95% for 10 min to rinse the column. Finally, B is lowered to 2% over 2 min and the column equilibrated for 20 min (170 min total run time). The mass range for MS scan was set to *m*/*z* 400−1250 and the MS/MS scan mass range was set to *m*/*z* 230−1500. A 250 ms survey scan (MS) was performed, and the top 50 ions were selected for subsequent MS/MS experiments employing an accumulation time of 65 ms per MS/MS experiment for a total cycle time of 3548s. Precursor ions were selected in high resolution mode (>30,000), tandem mass spectra were recorded in high sensitivity mode (resolution > 15,000). The selection criteria for parent ions included an intensity greater than 500 cps and a charge state ranging from + 2 to + 5. A 15s dynamic exclusion was used. The ions were fragmented in the collision cell using rolling collision energy, and collision energy spread (CES) was set to 5.

#### 2.9.1 Protein identification

The DDA MS raw files generated by the three runs were combined and subjected to database search in unison using ProteinPilot 4.5 software (AB SCIEX, USA) with the Paragon algorithm. The samples were input with the following parameters: iodoacetamide cysteine alkylation, digestion by trypsin, no special factors and biological modifications as ID focus. The searches were conducted through identification efforts in the UniProt Swiss‐Prot Human database (downloaded in January 2021, with 20,394 reviewed protein entries). A false discovery rate analysis was also performed.

#### 2.9.2 Bioinformatic analysis

The stand‐alone enrichment analysis tool FunRich (Functional Enrichment analysis tool; http://www.funrich.org) (Pathan et al., 2017) was used for performing enrichment analysis of site of expression on Funrich human background databases. The analysis of Gene Ontology (GO) non‐redundant terms and Reactome pathways was performed by using WebGestalt (Liao et al., 2019). Dataset of the HAFSC‐EVs proteins was analysed using Over‐Representation Analysis (ORA) by selecting the genome protein‐coding as reference set. All groups were significant with an FDR *<* 0.05.

### 2.10 RNA extraction

Total RNAs were isolated using TRIzol reagent (Life Technologies) according to the manufacturer's instructions. RNA quality was assessed by determining UV 260 nm absorbance at Qubit 3.0 Fluorometer (Thermo Scientific). RNA size distribution was analysed through RNA 6000 Nano LabChips (Agilent Technologies) processed on the Agilent 2100 Bioanalyzer (Agilent Technologies, Palo Alto, CA) using the total RNA electrophoresis program. Only RNAs with an RNA Integrity Number (RIN) ≥ 8 were used for subsequent analysis.

### 2.11 MiRNA analysis

For HAFSC‐EVs RNA analysis 300 ng of total RNA samples were labelled with FlashTag™ Biotin HSR Labelling kit following the manufacturer's instruction. The labelled RNAs were quantified, fractionated, and hybridized to the miRNA microarray (GeneChip™ miRNA 4.0 Array, Applied Biosystems™) according to the standard procedures provided by the manufacturer. RNA‐array hybridization was performed with agitation at 60 rotations per minute for 16–18 h using the Affymetrix GeneChip Hybridization Oven 645. The chips were washed and stained using a Genechip Fluidics Station 450 (Affymetrix). These were then scanned with an Affymetrix GeneChip Scanner 3000 (Affymetrix) to generate the raw data files (.CEL files). Probeset signals were pre‐processed and normalized using the Detection above background (DABG) and Robust Multichip Average (RMA) methods.

MiRNA expression profiles of HAFSC‐EVs (Table S1) were processed to keep only expressed miRNAs, which we conservatively considered to be those whose normalized expression values were greater or equal than the 25th percentile of the expression values of all miRNAs. We further dropped miRNAs not in common expressed in all HAFSC‐EVs and finally subjected the surviving miRNAs to the IPA's bioprofiling function with the aim to identify inflammation‐related diseases or biological functions. Inflammation‐associated miRNAs were wired and represented as a mixed phenotype‐miRNA network.

### 2.12 Lipidomic analysis

Cells and EVs samples were extracted with the one‐phase method MMC (Pellegrino et al., 2014). Samples were normalized during extraction by adding the appropriate volume of solvent mixture (2.5*10^^6^ cells in 1 mL of MMC). After adding solvents, each sample was well vortexed, then incubated for 30 min, 950 rpm, at room temperature. Samples were then centrifuged and 2 μL of supernatant were injected for LC‐MS analysis.

The LC‐MS system consisted in a Dionex UltiMate 3000 series (with binary pump, a thermostated autosampler and a column compartment) coupled with a Thermo Q‐exactive mass spectrometer (Thermo Fisher Scientific, Waltham, MA USA). Analyses were run with a reverse‐phase column Kinetex F5 (Phenomenex inc.), with a temperature of 45°C and a flow rate of 0.65 mL/min. Mobile phases consisted of 5 mM ammonium format and 0.1% formic acid in water (solvent A), and of 5 mM ammonium format and 0.1% formic acid in isopropanol (solvent B). A gradient elution of 28 min was used for lipid separation: time 0 min, solvent B 20%; time 3 min, solvent B 40%; time 16 min, solvent B 60%; time 16.5 min, solvent B 70%; time 24 min, solvent B 74%; time 28 min, solvent B 95%; and time 30, stop run. All solvents were purchased from Sigma‐Aldrich.

Mass spectrometry analysis was performed, as a first step, in positive/negative ion switching method in Full MS scan mode at high resolution. Regarding the criteria used for lipid identification these data have been processed with the Lipostar software (version 1.3.0, Molecular Discovery Ltd, UK) (Goracci et al., 2017) to perform a pre‐identification (by mass searching within a library of about 800,000 in silico fragmented lipids) of potential lipid species, for which a reduced number of selected samples was run again in order to obtain MS/MS data. The MS/MS data were then imported into the data matrix generated by Lipostar to perform the final lipid identification step, with exact mass match, retention time and MS/MS fragmentation.

### 2.13 HAFSC‐EVs uptake using confocal fluorescent microscopy

The ability of HAFSC‐EVs to communicate with dendritic cells was evaluated by confocal fluorescent microscopy in cDC Mutu. Dendritic cells (5 × 10^4^) were seeded on poly‐L‐Lysine‐coated glass cover slip and incubated with Dil labelled HAFSC‐EVs for 6 h at 37°C. After incubation, cells were washed twice with PBS, fixed for 20 min at room temperature with formaldehyde 4% and washed twice with PBS. Next, cells were stained with fluorescein isothiocyanate (FITC)‐labelled phalloidin (1:250) (Alexa Fluor™ 488 Phalloidin Thermo Fisher #A12379) for actin labelling. Imaging was carried out by Nikon Eclipse Te‐2000 coupled with a C1 confocal microscope. 60X plan apochromat oil immersion objective (Numeric Aperture: 1,4) was used. Experimental parameters were set out in order to collect the red and the green fluorescence using as excitation sources laser emitting at 543 nm and 488 nm, respectively. Photomultiper detectors (PMT) equipped with a long pass (LP) filter for wavelength longer than 570 nm (red fluorescence) and an interferential filter for 515 nm (green fluorescence) were used for fluorescence detection. ImageJ software ver.1.52n was used for post processing of the images and digital reconstruction of the 3D images by 3D‐projection of the Z‐stacking acquisitions (steps 150 nm). The images are representative of one out of three separate experiments.

### 2.14 HAFSC‐EVs uptake assay

HAFSC‐EVs uptake assays were also performed by flow cytometry. Labelled EVs were incubated with human PBMCs, murine splenocytes and isolated dendritic cells, as described previously, and after incubation, cells were washed twice with PBS and stained 30 min at 4°C with different antibodies mix: CD3, B220, Bst2, CD11c, MHCII, CD24, CD172, CD11b, B220, F480 and Ly6G to reveal the HAFSC‐EVs uptake in murine splenocytes immune compartments; CD3, CD19, HLADR, CD14, CD16, CD141, CD11c, CD1c, CD123 and Clec9a for HAFSC‐EVs uptake in human PBMCs; B220, Bst2, CD11c, MHCII, CD172, XCR1 antibodies (BioLegend) to evaluate the EVs uptake to different DC subsets. Next, cells were washed in MACS buffer and fixed in paraformaldehyde 1%. Data acquisition was performed by LSRFortessa (BD BioSciences) flow cytometer and analysed by FlowJo analysis software (Tree Star, OR, USA). For tSNE analysis, FCS files originated from analysed samples were loaded and analysed by using FlowJo vX software (BD, Biosciences). Samples were partitioned in different groups based on their treatment in vitro or in vivo. For each group including at least two samples, *n*: 1000 equal number of live CD45+ cells were concatenated in a single FCS file for in vivo study. For murine and human in vitro study at least n: 205000 equal number of live cells were concatenated in a single fcs file. A unique FCS file was used for the unbiased analysis, containing the three concatenated different group files. Data were partitioned and visualized after performing Dimensionality reduction using Optimized t‐distributed stochastic neighbour embedding (opt‐SNE) including the following parameters. Murine in vitro: CD24 PE‐Cy7, CD11c APC‐Cy7, MHCII BV510, CD172 Percp‐Cy5.5, CD11b BV421, F4/80 BV711, B220 BV786, CD3 APC, Bst2 BV650, EVs DiL. Human in vivo: CD19 FITC, CD1c PE‐Cy7, CD16 APC‐Cy7, CD11c BV510, CD123 Perc‐Cy5.5, CD141 APC, CLEC9A BV421, HLA‐DR BV786, CD14 BV711, CD3 BV605 and EVs DiL (Chen et al., 2022; Elashiry et al., 2020). Murine in vivo: CD8 FITC, Ly6G PE‐Cy7, CD11c APC‐Cy7, B220 BV510, CD45.2 Percp‐Cy5.5, Ly6C BV421, F4/80 BV711, CD11b AF700, MHCII PE‐CF594, CD3 APC, XCRI BV650, CD4 PE.

By using the same approaches, HAFSC‐EVs uptake assay was performed incubating EVs with mouse splenocytes and human PBMC cells previously treated with neutralizing antibodies anti‐ITGβ1 (Anti‐Mo/Rt CD29 #16‐0291‐85, eBioscience and ITGβ1 Monoclonal Antibody (TS2/16) #MA2910, ThermoFisher) and isotype control, respectively (Armerian Hamster IgG Isotype control #16‐4888‐81 and Mouse IgG1 kappa Isotype control #16‐4714‐82).

### 2.15 Intracellular cytokine staining

cDC2 from bone marrow were incubated over‐night with HAFSC‐EVs as previously described. The day after, 1 × 10^6^ conditionate cDC2 were activated with 50 ng/mL PMA (phorbol 12‐myristate 13‐acetate) (#P1585, Sigma‐Aldrich), 800 ng/mL ionomycin (#I9657, Sigma‐Aldrich) and 3 μg/mL BrefeldinA (#00‐4506‐51, Thermo Fisher) in complete IMDM for 4 h at 37°C to evaluate cytokines production by flow cytometry. After that, cDC2 were washed with PBS 1X and surface staining with rat anti‐CD16/32 (2.4G2) for 20 min at 4°C, for Fc receptors blocking. Next, cDC2 were labelled with: B220 BV510, CD11c AF700, MHCII BV421 and CD172 BV800 (Biolegend) antibodies in FACS buffer (1x PBS + 0.5% BSA and 2 mM EDTA) for 30 min at 4°C. Samples were fixed by incubation in 1x PBS + 2% (v/v) paraformaldehyde (#15714, Electron Microscopy Science) for 15 min at room temperature, followed by washes in MACS buffer, and permeabilized in MACS buffer + 0.5% (w/v) Saponin (#S7900, Sigma–Aldrich) for 5 min at room temperature. Cells were washed and cytokines IL‐6, IL‐12, IL‐23, and TNF‐*α* were stained in MACS buffer + 0.05% Saponin 30 min at 4°C. Isotype matched control antibodies were used for staining specificity. Cells were washed twice in MACS buffer + 0.5% (w/v) Saponin and resuspended in MACS buffer + 1% (v/v) PFA. Cytokines production were detected in CD172^+^ cells gated as follow: live cells, single cells, CD11c^+^B220^−^, CD11c^+^MHCII^+^ and CD172^+^ cells. Cells were analysed with FACS LSRFortessa (BD BioSciences) and data were analysed with FlowJo software (Tree Star).

### 2.16 Antigen presenting assay

2.5 × 10^4^ HAFSC‐EVs conditioned cDC2 or no conditioned cDC2 were cultured with 5×10^4^ CFSE labelled OT.II CD4^+^ T cells in complete media at the indicated concentrations of soluble OVA (#A5503, Sigma Aldrich). Cells were cultured at 37°C for 72 h and analysed by flow cytometry. OT‐II activation was evaluated as MFI of CD4^+^TCR‐Vα2^+^CD44^+^ cells. OT‐II proliferation was determined as the percentage of CD4^+^TCR‐Vα2^+^CD44^+^ cells which had undergone at least one CFSE dilution. OT‐II cells were gated as follow: live cells, single cells, TCR‐Vα2^+^, CD4^+^CD44^+^, CFSE^−^.

### 2.17 Treg induction by HAFSC‐EVs

BMDC2 were incubated over‐night with or without HAFSC‐EVs as previously described. The day after, conditionate and no conditionate DC2 were co‐cultures with naïve CD4^+^T cells in a ratio of 1:4 in 48 well/plate at 37°C for 3 days. Treg cells were determined by evaluating Foxp3 expression in T cells by intracellular staining and analysed by LSRFortessa (BD BioSciences) flow cytometer. Data were analysed by flowJo data analysis software.

### 2.18 EAE induction

Chronic EAE was induced as described (Crooks et al., 2017). C57BL/6 female mice received a subcutaneous (sc) immunization with 200 μg of myelin oligodendrocyte glycoprotein fragment MEVGWYRSPFSRVVHLYRNGK (MOG_35−55_ peptide; Cambridge Research Biochemicals) in incomplete Freund's adjuvant (Difco Laboratories), containing 4 mg/mL* Mycobacterium tuberculosis* TB H37 Ra (Difco Laboratories). Two hundred nanograms of pertussis toxin (List Biological Laboratories, Inc) in 200 μL PBS was injected intraperitoneally (i.p.) on the day of immunization and 2 days later. Groups of mice were injected with vehicle (PBS), vehicle conditioned cDC2 (cDC2‐PBS) and HAFSC‐EV conditioned cDC2 (cDC2‐EVs). 5 × 10^6^ of HAFSC‐EVs conditioned cDC2 and cDC2‐PBS were inoculated on day 5 and 14 after immunization. Mice were monitored daily, and neurological effects were scored using a 0–5 clinical standard scale 0 = no symptoms; 1 = limp tail; 2 = partial paralysis of hind limbs; 3 = complete paralysis of hind limbs or partial hind and front limb paralysis; 4 = tetraparalysis; 5 = moribund/death (Fallarino et al., 2010).

### 2.19 Real time PCR analysis

Lymphocytes from lymph nodes of EAE mice were cultured at a density of 2 × 10^6^/mL in IMDM medium containing 10% FCS in the presence of 25 μg/mL MOG35−55 or PBS as a control for 24 h. After incubation, RNA extraction and reverse transcription was performed according to manufacturer's protocol (TRIzolReagent #15596026, ThermoFisher Scientific; High‐Capacity cDNA Reverse Transcription Kit #4368813, Applied Biosysteem). Real‐time PCR for mouse IL‐17, IFN‐γ, *TGF‐β*, IL‐10 and *β‐*Actin was carried out with specific primers, reported in Table 1, using Brilliant SYBR Green QPCR Master Mix 2 × (Stratagene) with the Mx3000P qPCR System (Stratagene). Each sample was normalized to *β‐*Actin and values were determined by the relative quantification method (ΔΔCT) (mean ± SD of triplicate determinations).

**TABLE 1:** Primers sequence.

Gene	Sequences
*IL‐17*	Forward: GCTGACCCCTAAGAAACCCC
	Reverse: GAAGCAGTTTGGGACCCCTT
IFN‐γ	Forward: ATA TCT GGA GGA ACT GGC AA
	Reverse: CAT GAA TGC ATC CTT TTT CG
*TGF‐β*	Forward: CAC AGA GAA GAA CTG CTG TG
	Reverse: AGG AGC GCA CAA TCA TGT TG
*IL‐10*	Forward: ACC AGC TGG ACA ACA TAC TG
	Reverse: CGC ATC CTG AGG GTC TTC AG
*FOXP3*	Forward: CCC AGG AAA GAC AGC AAC CTT TT
	Reverse: TTC TCA CAA CCA GGC CAC TTG
*β‐*Actin	Forward: GGC TCC TAG CAC CAT GAA GA
	Reverse: AGC TCA GTA ACA GTC CGC C

### 2.20 Histology

On day 30 after MOG immunization, mice were sacrificed. Brains and spinal cords were isolated from sacrificed mice, fixed in 4% paraformaldehyde for 24 h and processed for paraffin histology. Histological staining with H&E was performed on 8‐μm‐thick transverse paraffin sections to reveal inflammatory infiltrates in the central nervous system. Luxol fast blue stain was performed on 8‐μm‐thick transverse paraffin sections to analyse the degree of demyelination.

### 2.21 Data source and gene expression analysis

The transcriptomics data utilized in this manuscript have been deposited in the NCBI Gene Expression Omnibus (GEO) at http://www.ncbi.nlm.nih.gov/geo. The accession codes for mouse splenocytes and human PBMC are GSE108097 and GSE107011, respectively. Digital expression matrix (dge) of Microwell‐seq data, derived from splenocytes of 6‐ to 10‐week‐old C57BL/6 mice, were acquired following the procedures outlined in a prior study (Han et al., 2018) and retrieved from the GEO database (GSM2906471). The Seurat package in R was used for subsequent analysis. Less than 200 genes per cell and cells with a mitochondrial content more than 5% were removed. Filtered data were normalized using a scaling factor of 10000, and the data were log transformed. The highly variable genes were selected using the FindVariableFeatures and data were scaled. Principal Component Analysis was performed using the top 1500 variable genes. Clustering was performed using the FindClusters function. TSNE was used to project cells into two dimensions using 10 first principal components. We re‐clustered cell populations and performed normalization, found variable genes and performed PCA, TSNE and clustering as described above. We used the pheatmap package to generate a heatmap with the expression of *Itgb1* and *Itgb3*. The values of transcripts per million of the average gene expression by clusters were log‐transformed (log2(TPM+1)). Differential Gene Expression analysis between clusters were performed using FindMarkers function and DESeq2 test. To perform analysis on PBMC data, we used the bulk RNA‐Seq dataset of purified blood cell subtypes, available from GSE107011 (Monaco et al., 2019) containing expression profiles for 29 blood cell subtypes, including T and B cells, pDC, cDC, classical (cMono), intermediate (intMono) and non‐classical (ncMono) monocytes. We downloaded the expression matrix normalized by TPM, selected the populations of interest depicted them in a heatmap using the log2(TPM + 1) transformation. All the data processing was carried out in the R environment.

### 2.22 Statistical analysis

EVs were purified from three different stem cell lines obtained from the amniotic fluid of pregnant women at 16–17 weeks (aged 35–40 years) undergoing amniocentesis as part of routine prenatal diagnosis. EVs of the respective stem cell lines were used for exosome characterization. For the in vitro and in vivo study, we used EVs derived from the three cell lines. Protein quantification by the maximum absorbance at 280 nm was used to normalize the protein content of the EVs samples for DC treatment. Data are expressed as the means ± SD. Differences between groups were analysed using one or two‐way ANOVA following by multiple comparison tests. All statistical analyses were performed using GraphPad Prism 9 software (GraphPad). Differences were considered statistically significant when *p* values were < than 0.05. Statistical analysis of proteomic and miRNA data are described in the respective methods.

## 3 RESULTS

### 3.1 HAFSC‐EVs isolation and characterization

**FIGURE 1:**
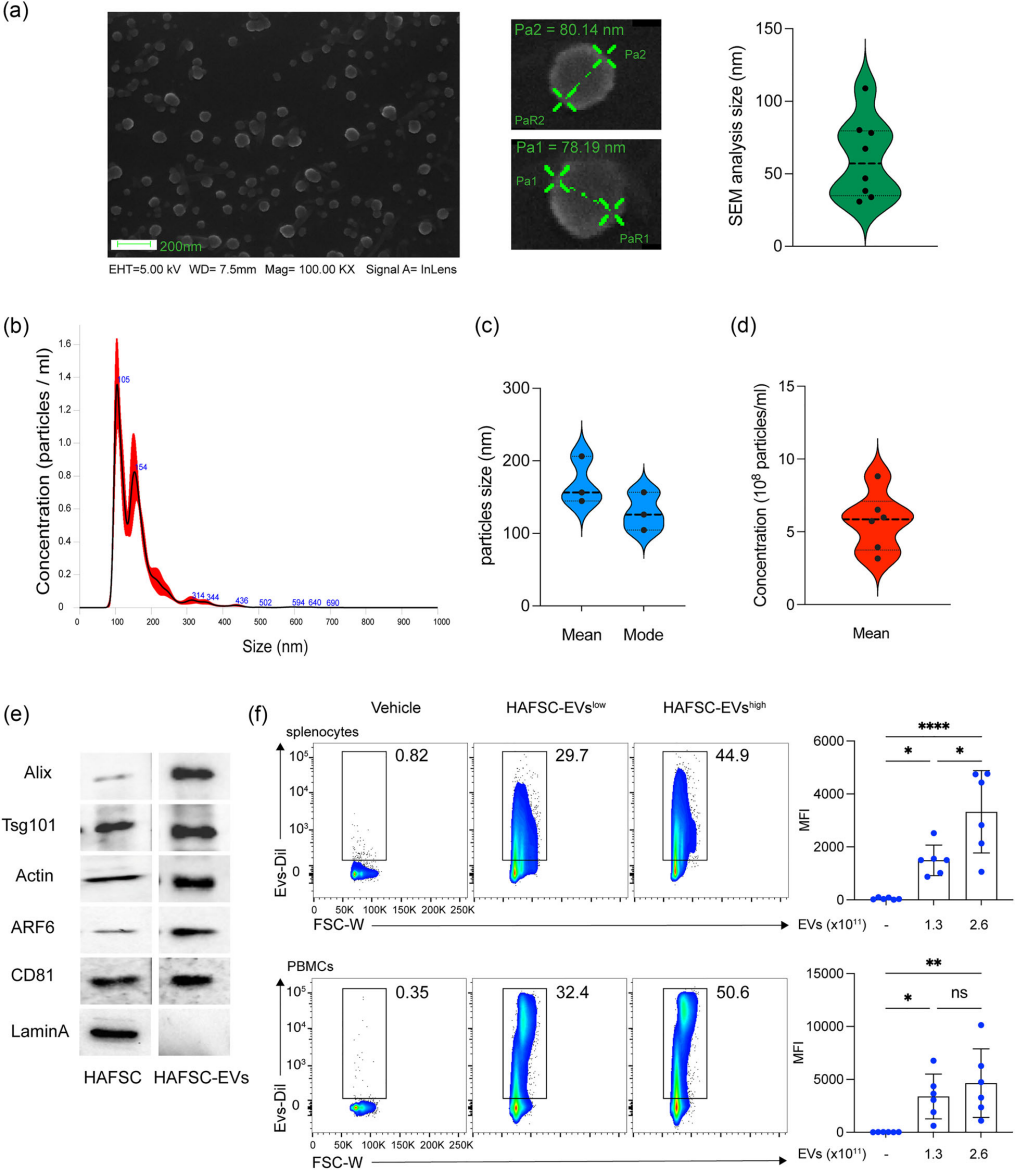
Characterization of EVs derived from human amniotic fluid stem cells (HAFSC‐EVs). (a) HAFSC‐EVs morphology and diameter measure by SEM. Magnification bar corresponds to 200 nm; Violin box graph displaying mean sizes of HAFSC‐EVs. (b) HAFSC‐EVs size distribution and concentration determinated by nanoparticle tracking analysis. Representative spectra are shown. Blue values correspond to the size of the main peaks in the histogram. (c) Mean and mode of particles size (nm) and (d) mean concentration of HAFSC‐EVs (number/mL) are reported in the graphs. (e) Molecular characterization of isolated HAFSC‐EVs by Western blot of specific EV markers compared to parent stem cells. Western blot analysis revealed the presence of CD81, ARF6, Actin, Tsg101, Alix and absence of Lamin‐A that is expressed only by cells. (f) Representation plot of EVs binding on murine splenocytes and human PBMCs after 6 h exposition of cells with EVs (HAFSC‐EVs^low^ and HAFSC‐EVs^high^ correspond to 1.3 × 10^9^ and 2.6 × 10^9^ particles/1 × 10^6^ cells). Data are represented as mean ± SD of MFI (mean fluorescent intensity). *p < 0.05; **p < 0.01 by one‐way ANOVA with Tukey's multiple comparison test.

In an effort to better understand the mechanisms underlying the immunoregulatory properties of EVs, we investigated the direct influence of HASFC‐EVs on the inflammatory potential of antigen‐presenting cell subsets. Specifically, we isolated EVs from human amniotic fluid‐derived stem cells cultured in serum‐free medium to avoid contamination from serum‐derived EVs (Mezzasoma et al., 2022). We confirmed that these culture conditions did not affect either the expression of stem cell markers or the number of live stem cells (Figure S1A, B). Isolation of EVs from HAFSC‐conditioned cell medium was achieved through differential ultracentrifugation (Figure S1C). The EVs were further characterized by scanning electron microscopy (SEM) to obtain high‐resolution and magnified images and Transmission Electron microscopy (TEM) (Figure S1D). As expected, SEM observation revealed vesicles of spherical shape with an average diameter of 75 ± 3 nm and, compatible with exosome size (Figure 1a). The distribution plots obtained from nanoparticle tracking analysis (NTA) indicated that the average diameter of the vesicles was approximately 150 nm (Figure 1b, c), which is larger than the size observed through SEM, suggesting the presence of aggregates. Furthermore, the yield of HAFSC‐EVs per isolation was found to range from 4 × 10^8^ to 5 × 10^8^ particles/mL, influenced by the HAFSC culture conditions (Figure 1d). The Western blot analysis provided further confirmation that the isolated fraction contained highly enriched extracellular vesicles and exosome markers (available on http://www.exocarta.org/), including ALIX, ARF6, CD81, TSG101, and β‐actin (Figure 1e).

EVs are capable of fusing directly with the plasma membrane of target cells (Montecalvo et al., 2012; Obregon et al., 2006). This allows membrane‐encapsulated cargoes to be transferred from donor to acceptor cells, potentially impacting the functional phenotype of the latter (Skog et al., 2008; Valadi et al., 2007). Additionally, recent years have seen the demonstration of the essential and ubiquitous involvement of EVs in fundamental immune mechanisms and immune‐mediated disease processes (Buzas, 2023).

Therefore, to identify immune cells able to take up HASFC‐EVs, we stained EVs with Dil fluorescent dye a carbocyanine derivative that can be embedded into the membrane of EVs in a noncovalent manner allowing EVs labelling, tracking and imaging (Wiklander et al., 2015) (Figure S1E). Two different amounts of Dil‐labelled HASFC‐EVs were then incubated for 6 h with total human PMBCs or murine splenocytes, that were assessed for Dil expression by cytofluorimetric analysis. Our study revealed that incubation of human and murine cells with the two different EVs amounts led to a significant dose‐dependent increase in Dil signal (Figure 1f).

Overall, our data showed that HAFSCs produce large numbers of EVs that are quite homogeneous in size, express EV‐specific markers, and possess the ability to interact with both human and murine immune cell targets.

### 3.2 HAFSC‐EVs are preferentially taken up by conventional type‐2 cDCs

Previous studies have demonstrated the crucial role of EVs derived from amniotic fluid as modulators of immune functions (Del Rivero et al., 2022; Mezzasoma et al., 2022; Romani et al., 2015). To identify the specific immune cells capable of taking up HAFSC‐EVs, we incubated Dil‐labelled HAFSC‐EVs with human PBMCs or mouse splenocytes for 6 h. We then assessed Dil expression in selected immune cell types by flow cytometry using a combination of specific antibodies reacting to cell‐specific markers.

**FIGURE 2:**
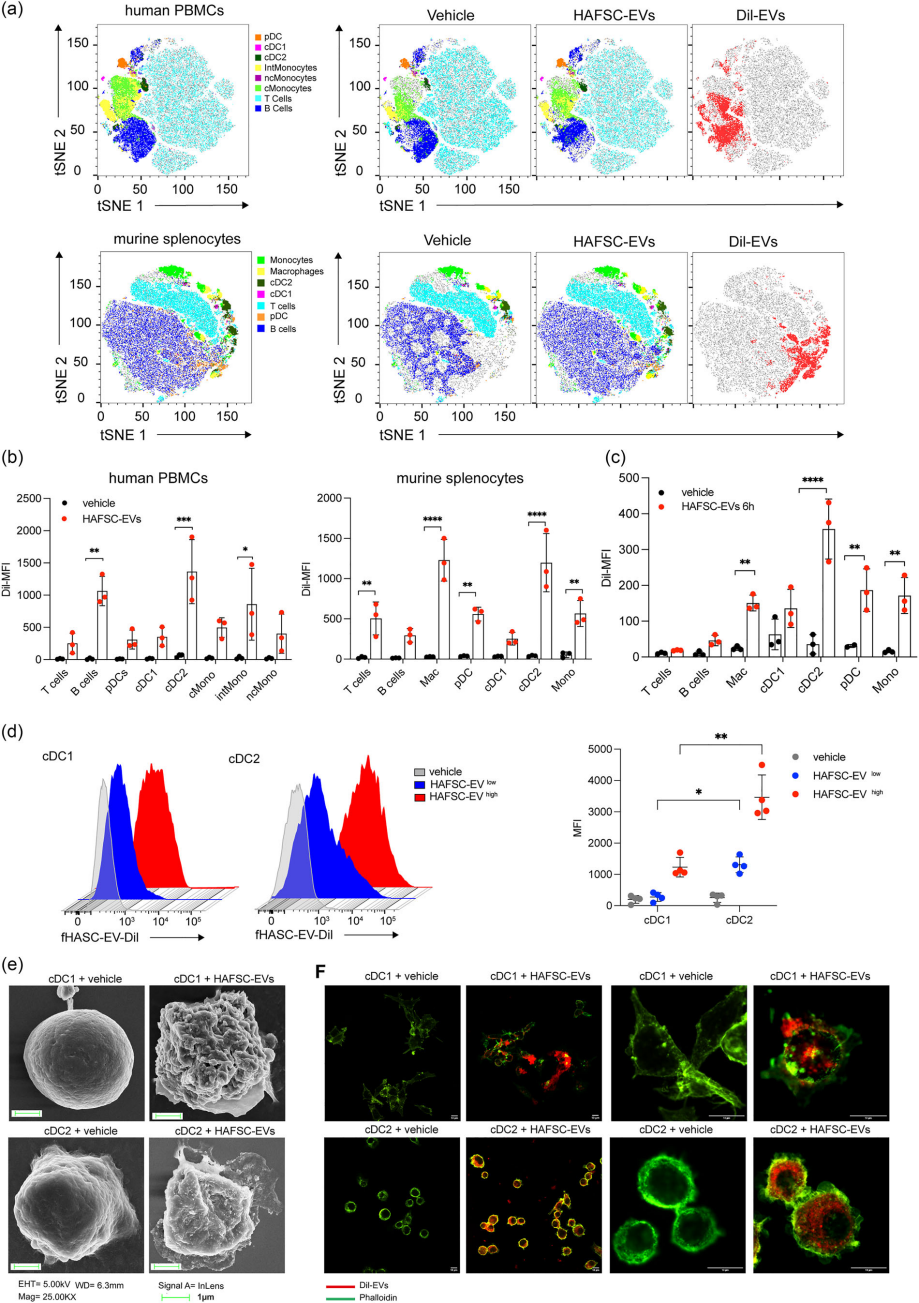
HAFSC‐EVs uptake in immune cells. (a) Total hPBMC and murine splenocytes were treated in vitro with Dil‐HAFSC‐EV 1.3 × 10^9^ (HAFSC‐EV^low^) for 6 h, washed with PBS, stained with the fluorophore‐conjugated antibodies and analysed by flow cytometry. Two‐dimensional t‐SNE analysis (FlowJo) represents the various cell populations before and after treatment with HAFSC‐EVs and the distribution of EVs (red) on total human PBMCs and murine splenocytes (grey). Data shown are representative of three independent experiments. (b) Dil signal was registered in different immune cell populations by using antibodies directed against specific immune cell markers and analysed by Flow Jo. The most significant EVs uptake was detected in cDC2 cells both in human and murine samples. Data are represented as mean ± SD of MFI (mean fluorescent intensity) of three experiments (**p < 0.01, ***p < 0.001, ****p < 0.0001 two‐way ANOVA with Bonferroni's multiple comparison test). (c) Dil‐HAFSC‐EV or vehicle were injected intravenously into mice, and spleens were harvested after 24 h (see also in Figure S2). Dil signal was evaluated *ex‐vivo* by flow cytometry in different immune cell populations. Data are represented as mean ± SD of MFI (mean fluorescent intensity) of three independent experiments (****p < 0.0001 two‐way ANOVA with Bonferroni's multiple comparison test). (d) Vesicles uptake was measured in bone marrow derived DCs (cDC1 and cDC2) after 6 h incubation with 1.3 × 10^9^ (HAFSC‐EVs^low^) and 2.6 × 10^9^ (HAFSC‐EVs^high^) Dil‐HAFSC‐EVs /1 × 10^6^ cells. Indicated is the mean ± SD of MFI of cDC1 and cDC2, detected by anti‐CD24 and anti‐CD172, respectively, positive for Dil‐HAFSC‐EVs (showed histograms is one experiment representative of three) (*p < 0.05 **p < 0.001 two‐way ANOVA with Tukey recommended multiple comparison test). (e) Vesicles uptake by cDC1 and cDC2 derived from bone marrow analysed by SEM (scanning electron microscopy) after 6 h EVs treatment. Representative images of three different experiments. Magnification bar corresponds to 1 μm. (f) Confocal immunofluorescence images of cDC1 and cDC2 cells treated with Dil‐labelled‐HAFSC‐EVs for 6 hours, controls were treated with vehicle. Pictures show representative confocal fluorescence images of three independent experiments. Magnification bar corresponds to 10 μm.

tSNE analysis showed the distribution of EVs, indicated by Dil expression in red, throughout different cell populations in total human PBMCs and murine splenocytes (Figures 2a and S2A).

Furthermore, the analysis conducted by flow cytometry confirmed that Dil^+^ cells in both human PBMCs and mouse splenocytes were predominantly represented by cell types expressing myeloid markers such as cDCs, macrophages, and pDCs, rather than T cells (Figures 2b and S2B). Surprisingly, we found that cDC2 exhibited a higher Dil expression than cDC1, which prompted us to further investigate whether EVs were preferentially internalized by cDC2 subtype. To confirm these data, we performed in vivo experiments. Specifically, naïve C57BL/6 mice were intravenously injected with Dil‐labelled HAFSC‐EVs, whereas control mice received vehicle (PBS) and Dil‐expressing cells were recorded at 6 and 24 h. in vivo, we also found a higher uptake of HAFSC‐EVs by cDC2 in the spleen compared with cDC1. We also confirmed minimal uptake by other immune cell types, such as T, B lymphocytes and pDCs at both time points (Figures 2c and S2C). In addition, 24 h after injection, we observed uptake of HAFSC‐EVs in CD45^+^ cells in other regions such as brain, lung, and liver (Figure S2D).

To further confirm the preference for EV uptake by mouse cDC2 compared with cDC1, we differentiated cDC subsets from bone marrow of wild‐type C57BL/6 mice (Figure S2E) and treated them separately with Dil‐labelled HAFSC‐EVs or vehicle (PBS) for 6 h. We then used flow cytometry, SEM, and confocal microscopy to visualize the results. Flow cytometry showed that cDC2 preferentially captured HAFSC‐EVs compared with cDC1 (Figure 2d). SEM revealed the presence of vesicular structures outside the dendritic cell membrane for both cDC subsets, indicating that both types of cDCs were able to interact with EVs (Figure 2e). However, confocal microscopy analysis showed that cDC2 exhibited higher efficiency in internalizing HAFSC‐EVs compared with cDC1 (Figure 2f and Videos S1 and S2).

These findings confirm that while HAFSC‐EVs interact with both cDC subsets, but they are more effectively internalized into the intracellular compartments of cDC2 than of cDC1. Based on these results, subsequent studies investigated the specific effects of HAFSC‐EVs on the cDC2 subset.

### 3.3 ITGβ1 blockade inhibits uptake of HAFSC‐EVs by cDC2

Integrins are a central mechanism for cells to interact with and sense their extracellular environment. Integrins ITGβ1 (CD29) and ITGβ3 (CD61) have been shown to be involved in EVs uptake into cells (Nguyen et al., 2021; Shin et al., 2020). In particular, the integrin CD29 is capable of binding multiple targets, including CD81 (Hazawa et al., 2014), which is expressed on the surface of EVs. To investigate the mechanism underlying the uptake of HAFSC‐EVs by cDC2 cells, we first examined CD29 expression on mouse and human immune cells.

**FIGURE 3:**
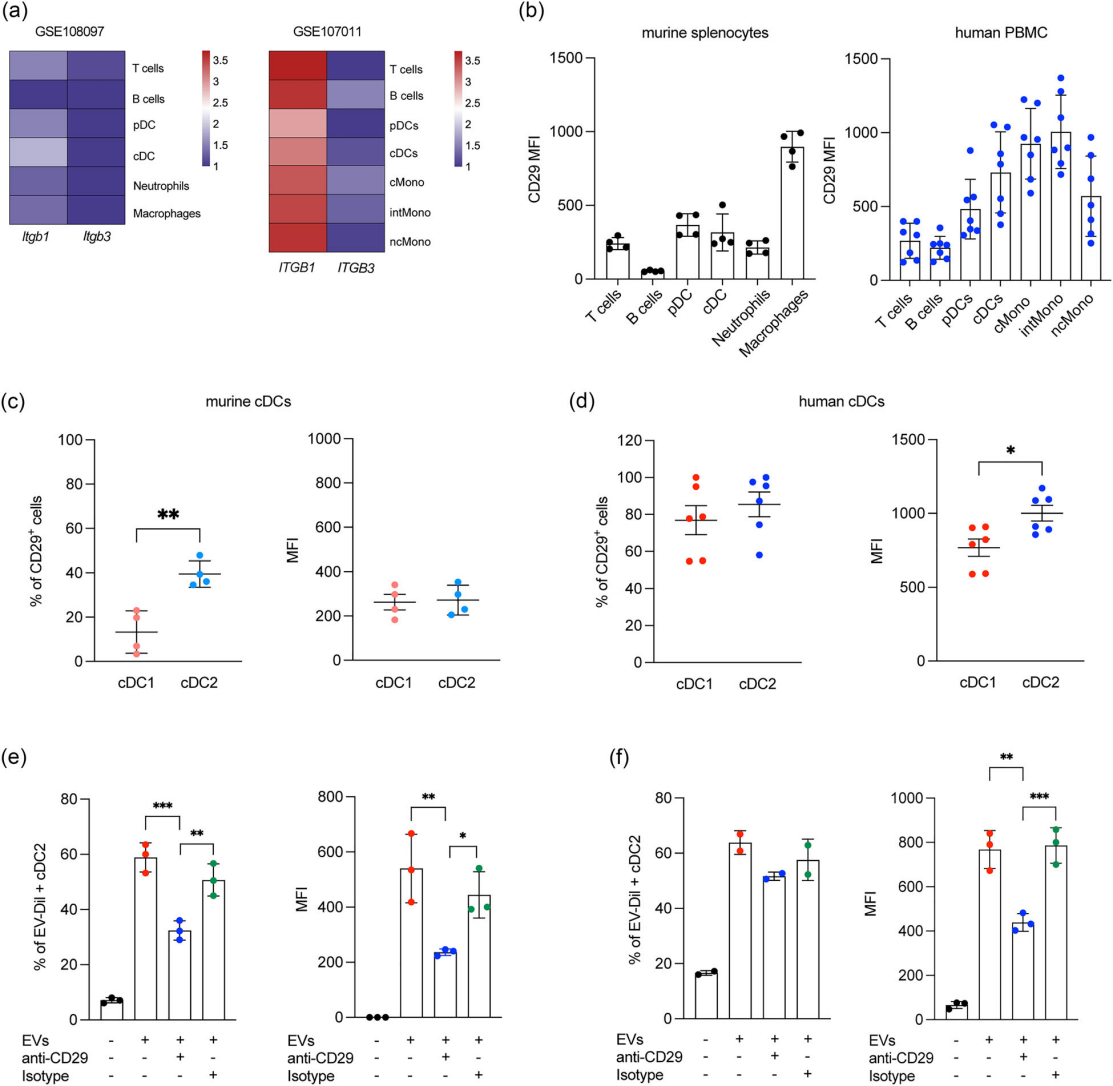
Integrin CD29 mediates HAFSC‐EVs trafficking to cDC2. (a) Heatmap displaying gene expression of *ITGB1* and *ITGB3* across diverse immune cell populations in mouse splenocytes (GSE108097) and human PBMC (GSE107011). Log‐transformed transcripts per million (log2(TPM+1)) values were used. Colour scale indicates relative expression levels (red for higher, blue for lower). (b) Itgß1 protein expression on immune cells by FACS analysis on total murine splenocytes (*n* = 4) and human PBMC (*n* = 7) reported as MFI ± SD. (c, d) CD29 expression by murine and human cDC1 and cDC2 reported as percentage of CD29 positive cells and as MFI ± SD (*p < 0.05 **p < 0.005, unpaired *t*‐test, two‐tailed). (e, f) Flow cytometry analysis of EVs‐Dil^+^ murine and human cDC2 after treatment with CD29 neutralizing or Isotype antibody (as control). Data are shown as mean ± SD of MFI or percentage of Dil^+^cDC2 (n = 3, *p < 0.05; **p < 0.01, ***p < 0.001 by one‐way ANOVA with Bonferroni's multiple comparison test).

Data analysis from online available datasets showed that ITGß1 is more highly expressed than ITGß3 in immune cells and especially in macrophages and cDCs in both human PBMCs and mouse splenocytes (Figure 3a) and (Figure S3). Based on these data, we focused on the expression of ITGß1 protein and we confirmed that it is highly expressed in cDCs and macrophages in both human PBMCs and mouse splenocytes (Figure 3b). We then examined whether there was differential CD29 expression between the two subsets of cDCs. We found a higher percentage of CD29‐expressing mouse cDC2 cells compared with cDC1, and in human PBMCs, we detected higher CD29 expression (MFI) in cDC2 compared with cDC1 (Figure 3c, d).

To investigate the role of CD29 in the uptake of HAFSC‐EVs by cDC2 cells, we performed experiments with neutralizing antibodies specific for mouse and human CD29. Total mouse splenocytes and human PBMCs were cultured in the presence of these antibodies, whereas equivalent isotype antibodies were used as controls. Cells were then incubated with Dil‐labelled HAFSC‐EVs for 6 h. Our results showed that blocking murine and human CD29 significantly inhibited the uptake of HAFSC‐EVs by cDC2 cells in both murine (Figure 3f) and human samples (Figure 3g). In conclusion, our results provide evidence for the involvement of CD29 integrin in the uptake of HAFSC‐EVs by cDC2 cells.

### 3.4 Immunoregulatory proteins and miRNA are enriched in HAFSC‐EVs

To gain deeper insight into the biological effects of EVs on cDC2 cells, we performed extensive compositional and characterization analyses of HAFSC EVs. We first focused on the lipid composition of HAFSCs and their released EVs, as lipids are known to play a critical role in membrane fusion and selective cellular uptake. Our lipidomic analysis revealed the presence of glycerolipids, glycerophospholipids, sterols, and sphingolipids in both HAFSCs and their EVs.

**FIGURE 4:**
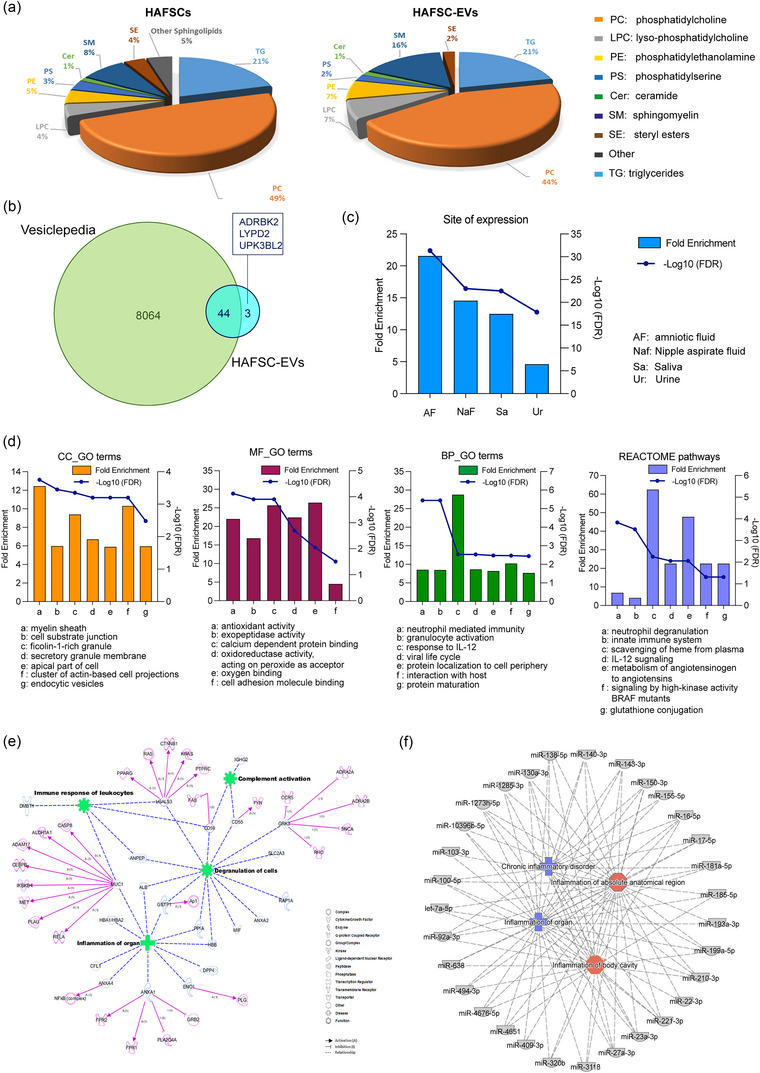
Analysis of HAFSC‐EVs composition. (a) Comparison of lipids composition of HAFSCs and HAFSC‐EVs membrane. Relative abundances for each lipid classes in cells and EVs: TG Triacylglycerols, PC Glycerophosphocholines, LPC Monoacylglycerophosphocholines, PE Glycerophosphoethanolamines, PS Glycerophosphoserines, Cer Ceramides, SM Sphingomyelins, SE Sterol Lipids, Other Sphingolipids. (b) The Venn diagram of proteins identified in HAFSC‐EVs and EVs isolated from biofluids (amniotic fluid, aqueous humour, plasma, serum, saliva, urine) and placenta. (c) Enrichment analysis of site of expression of proteins identified in the HAFSC‐EVs performed by Funrich. (d) Gene Ontology terms and Reactome Pathways enrichment analyses performed by WebGestalt. Among the 10 most enriched functional terms listed in the Table S1 sheet “*Enriched CC_GO TERMS*”, the first seven are reported in the histograms. Within the MF_GO terms, only six terms were found significantly enriched. CC: Cell Component; BP: Biological Process; MF: Molecular Function. (e) Network generated in Ingenuity Pathway Analysis (IPA) for proteins identified in HAFSC‐EVs. The meaning of the shapes (nodes) and type of interactions (edges) are defined in the graphical legends. Blu nodes are proteins found in HAFSC‐EVs; green nodes indicate functional activities in which HAFSC‐EVs proteins are involved; pink nodes are predicted direct targets of HAFSC‐EVs proteins. (f) Mixed phenotype‐miRNAs network generated by Ingenuity Pathway Analysis (IPA) for miRNAs identified in HAFSC‐EVs. 31 miRNAs were associated to inflammation; blue nodes are associated with chronic inflammatory disorders while red nodes are implicated in inflammation of absolute anatomical region or body cavity.

In particular, the analysis revealed that glycerolipids in both cells and EVs consisted of triacylglycerols, whereas glycerophospholipids were mainly glycerophosphocholines (including monoacylglycerophosphocholines), followed by glycerophosphoethanolamines and glycerophosphoserols. Among sphingolipids, sphingomyelins were the major component, with a small amount of ceramides detected. Cholesterol and steryl esters were also detected in both HAFSCs and their EVs (Figure 4a). While the quality of these lipids was similar in HAFSCs and their EVs (Figure 4a), there were differences in the enrichment of certain lipid species between HAFSCs and their EVs. In particular, we found a higher proportion of sphingomyelin and lyso‐phosphatidylcholine in EVs compared to HAFSCs, indicating a distinct lipid enrichment in EV populations (Figure 4a). These results suggest that HAFSC‐EVs also possess unique lipid composition that may contribute to their functional properties and interactions with target cells.

We then performed proteomic analysis of the HAFSC‐EVs to gain insight into their protein composition. This analysis led to the identification of 47 major proteins listed in Table S2 (sheet *“HAFSC‐EVs Protein ID”*). To determine whether these proteins are also present in other EVs, we compared our results with the Vesiclepedia database using FunRich software (http://www.funrich.org). The Venn diagram in Figure 4(b) shows that of the 47 proteins identified in HAFSC EVs, 44 have already been found in EVs from various biofluids, including amniotic fluid, aqueous humour, plasma, serum, saliva, urine, and placenta. Of note, three proteins, namely, ADRBK2, LYPD2, and UPK3BL2 (Table S2), had not yet been documented in the Vesiclepedia database.

In addition, the enrichment analysis revealed that most of the proteins identified in the HAFSC‐EVs were secreted in biofluids. Remarkably, the analysis indicated a significantly higher fold enrichment (FE: 22.043; FDR: 1.576E‐32) for proteins expressed in amniotic fluid (Figure 4c and “*FunRich Site of Expression*” sheet in Table S2). Furthermore, the Reactome pathway analysis revealed that proteins identified in HAFSC‐EVs exhibited enrichment in functional classes associated with the membrane compartment and secretory granules (Figure 4d and the “Enriched CC_GO TERMS” sheet in Table S2). Moreover, these proteins displayed activities particularly related to exopeptidase and antioxidant functions (Figure 4d histogram “*MF_GO terms*” and the “*Enriched MF_GO TERMS*” sheet in Table S2), as well as involvement in various immune system activities (Figure 4d histogram “*BP_GO terms*,” Figure 4d REACTOME pathways, the “*Enriched BP_GO TERMS*,” and “*Enriched REACTOME pathways*” sheets in Table S2).

Network analysis performed using Ingenuity Pathway Analysis (IPA) revealed that more than 22 of the 47 proteins identified in HAFSC‐EVs (approximately 45%) were associated with immune system function and disease status (Figure 4e). These proteins have been reported to affect multiple targets involved in the regulation of immune cell function. Among those MUC1 and ANXA4; Formyl Peptide Receptors 1 and 2 (FPR1 and FPR2) which are involved in anti‐inflammatory processes (Caso et al., 2021); cytosolic phospholipase A2 (PLA2G4A) which plays a crucial role in inflammatory processes and is inhibited by ANXA1, well known to exert protective and anti‐inflammatory actions in several disease models (Caso et al., 2021); GSK3, whose role in modulating immune cells is recently emerging (Grozdanov & Danzer, 2020). Altogether, these observations suggest that proteins delivered by HAFSC‐EVs have the potential to modulate the activities of resident proteins in target cells, which could lead to a new balance between pro‐ and anti‐inflammatory responses.

Another class of biological molecules involved in EVs cell‐to‐cell communication and regulation is characterized by microRNA (miRNA). We conducted miRNA expression profiling studies in HAFSC‐EVs and their potential functional role. Importantly, we found that 337 miRNAs co‐expressed among HAFSC‐EVs and that 31 of them were functionally associated with inflammation. Of note, we found miR‐155‐5p, which is known to play diverse roles in immune cell regulation, including modulating the activation and function of various immune cells such as T cells, B cells, and macrophages (Duan et al., 2016; Foster et al., 2013), and miR‐181a that has been shown to play a role in regulating T‐cell responses, including T‐cell activation and differentiation and also linked to the endothelial inflammation via regulating critical signalling pathways, such as downstream NF‐κB (Sun et al., 2014) (Figure 4f).

These results suggest that cDC2 may acquire several proteins and RNA involved in immune regulation through the uptake of HASFC‐EVs, possibly contributing to the reprogramming of the immunophenotype of this specific DC subset.

### 3.5 HAFSC‐EVs convert inflammatory cDC2 to tolerogenic cDCs

One of the major functions attributed to EVs from various sources is their ability to modulate APCs to produce specific cytokines and to activate T cells for either effector or regulatory functions (Akbar et al., 2021).

**FIGURE 5:**
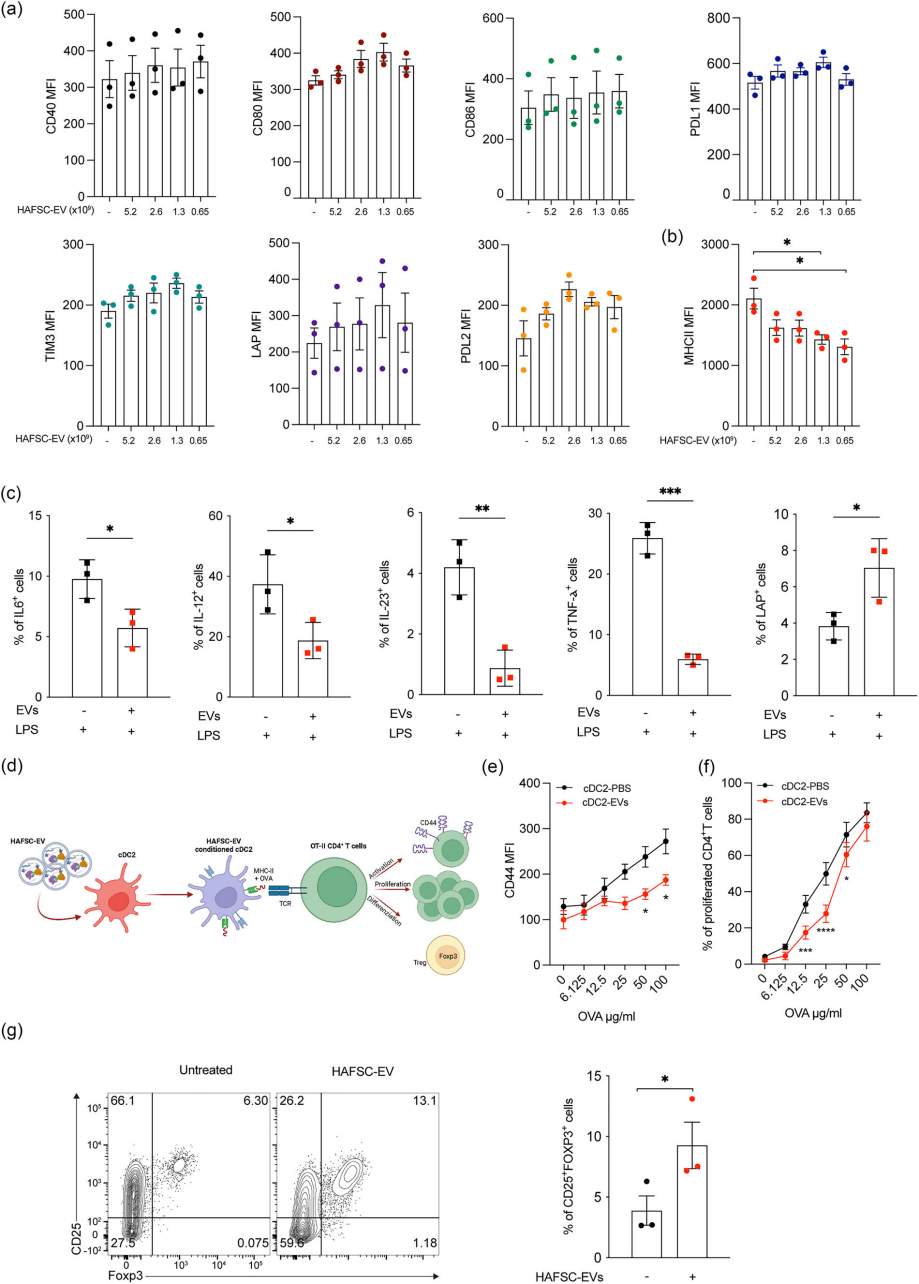
cDC2 conditioning by HAFSC‐EVs treatment. (a, b) Analysis of cDC2 activation markers was performed by FACS by overnight incubation with various amounts of HAFSC‐EVs as shown. Data are shown as mean ± SD of MFI (*n* = 3, *p < 0.05; by one‐way ANOVA with Tukey recommended multiple comparison test). (c) Analysis of cytokines produced by bone marrow derived cDC2 pre‐treated overnight with HAFSC‐EVs and subsequently treated with LPS (1 ug/mL) for 6 h in the presence of PMA/Ionomycin and Brefeldin‐A. Percentage of cells positive for IL‐6, IL‐12, IL‐23, TNF‐α, and LAP are shown as mean ± SD (*n* = 3, *p < 0.05; ***p < 0.001 by unpaired *t*‐test, two‐tailed). (d) Schematic representation of co‐culture characteristics between EVs‐conditionated‐cDC2 and mouse CD4^+^ T cells. (e) OT.II CD4^+^ T cells were co‐cultured with cDC2 pretreated with HAFSC‐EVs^low^ overnight as indicated in D, in the presence of different concentrations of soluble OVA protein. Data are shown as MFI (*n* = 3, *p < 0.05; by two‐way ANOVA with Bonferroni's multiple comparison test). (f) cDC2, treated as in D, were assayed for presentation to CFSE‐labelled OT‐II T cells in the presence of different concentrations of soluble OVA protein. Data are shown as percentage of CFSE^−^CD4^+^CD44^+^ TCRVα2^+^ as mean ± SD (*n* = 3, *p < 0.05; ***p < 0.001, ****p < 0.0001, two‐way ANOVA with Bonferroni's multiple comparison test). (g) CD4^+^ T cells were purified from the spleen of wild type mice and co‐cultured with cDC2 pre‐treated with HAFSC‐EVs^low^. Three days after Foxp3 expression was evaluated by flow cytometry. Data are shown as mean ± SD of percentage of CD25^+^Foxp3^+^ cells (*n* = 3, *p < 0.05; by unpaired *t*‐test, two‐tailed). Dots represent each individual mouse.

To investigate the effects of HAFSC‐EVs on cDC2 cells, we examined the expression of DC activation markers, including CD80, CD86, and CD40, as well as inhibitory molecules commonly found on tolerogenic DCs, such as LAP and PDL1. We treated cDC2 cells with different amounts of HAFSC‐EVs for 24 h and evaluated the activation marker expression. Surprisingly, we observed no significant changes in the expression of CD80, CD86, CD40, LAP, or PDL1 upon treatment with HAFSC‐EVs (Figure 5a). However, we observed a significant decrease in the levels of major histocompatibility complex class II (MHCII) after treatment with HAFSC‐EVs when used at low amounts (Figure 5b). These findings suggest that HAFSC‐EVs specifically impact MHCII expression in cDC2 cells without significantly altering the expression of other activation or inhibitory markers commonly associated with DC function.

To further investigate the impact of HAFSC‐EVs on cDC2 function, we performed cytokine analyses on sorted mouse cDC2 cells treated with LPS after pretreatment with HAFSC‐EVs or vehicle. Intriguingly, we observed a significant decrease in the production of IL‐6, IL‐12, IL‐23 and TNF‐α cytokines by cDC2 cells following pretreatment with HAFSC‐EVs under inflammatory conditions (Figure 5c). In contrast, treating cDC2 cells with HAFSC‐EVs increased the percentage of LAP‐expressing cells compared to cells treated with LPS alone. (Figure 5c).

Conventional type 2 DC also control the balance between immunity and tolerance through their ability to activate naïve CD4^+^ T cells (Audiger et al., 2017). To further explore the functional capabilities of HAFSC‐EV‐conditioned cDC2 (HAFSC‐EV‐cDC2), we examined their ability to activate naive OT.II CD4^+^ T cells. In T‐cDC co‐cultures (Figure 5d), we observed that overnight treatment of cDC2 cells with HAFSC‐EVs, before ovalbumin (OVA) antigen exposure, significantly reduced proliferation and activation of T cells (Figure 5e, f).

Remarkably, we also observed a higher proportion of OT.II cells expressing the regulatory T cell marker Foxp3 after treatment of cDC2 cells with HAFSC‐EVs compared with vehicle‐treated control cells (Figure 5g).

These results highlight the ability of HAFSC‐EVs to modulate the cDC2's cytokine profile, leading to a reduction in the production of pro‐inflammatory cytokines. Additionally, the results indicate that HAFSC‐EVs promote potentially tolerogenic properties in cDC2 cells. Furthermore, these data suggest that HAFSC‐EVs can modulate the priming capacity of cDC2 cells, resulting in decreased T‐cell activation and increased polarization toward regulatory T cells.

### 3.6 Decreased EAE severity through HAFSC‐EVs‐conditioned cDC2 cells

Inflammatory cDCs have been identified as a significant source of proinflammatory cytokines, including IL‐6, which plays a critical role in priming pathogenic Th17 cells in mice with experimental autoimmune encephalomyelitis (EAE) (Heink et al., 2017; Mundt et al., 2019). Additional studies have shown that the adoptive transfer of tolerogenic dendritic cell subsets can effectively inhibit proliferation of antigen‐specific pathogenic T cells, induce regulatory T cells (Tregs), and suppress EAE (Awasthi et al., 2007; Li et al., 2008). In addition, our recent data have shown that mice lacking certain cDC2 subsets are protected from developing EAE (Gargaro et al., 2022).

**FIGURE 6:**
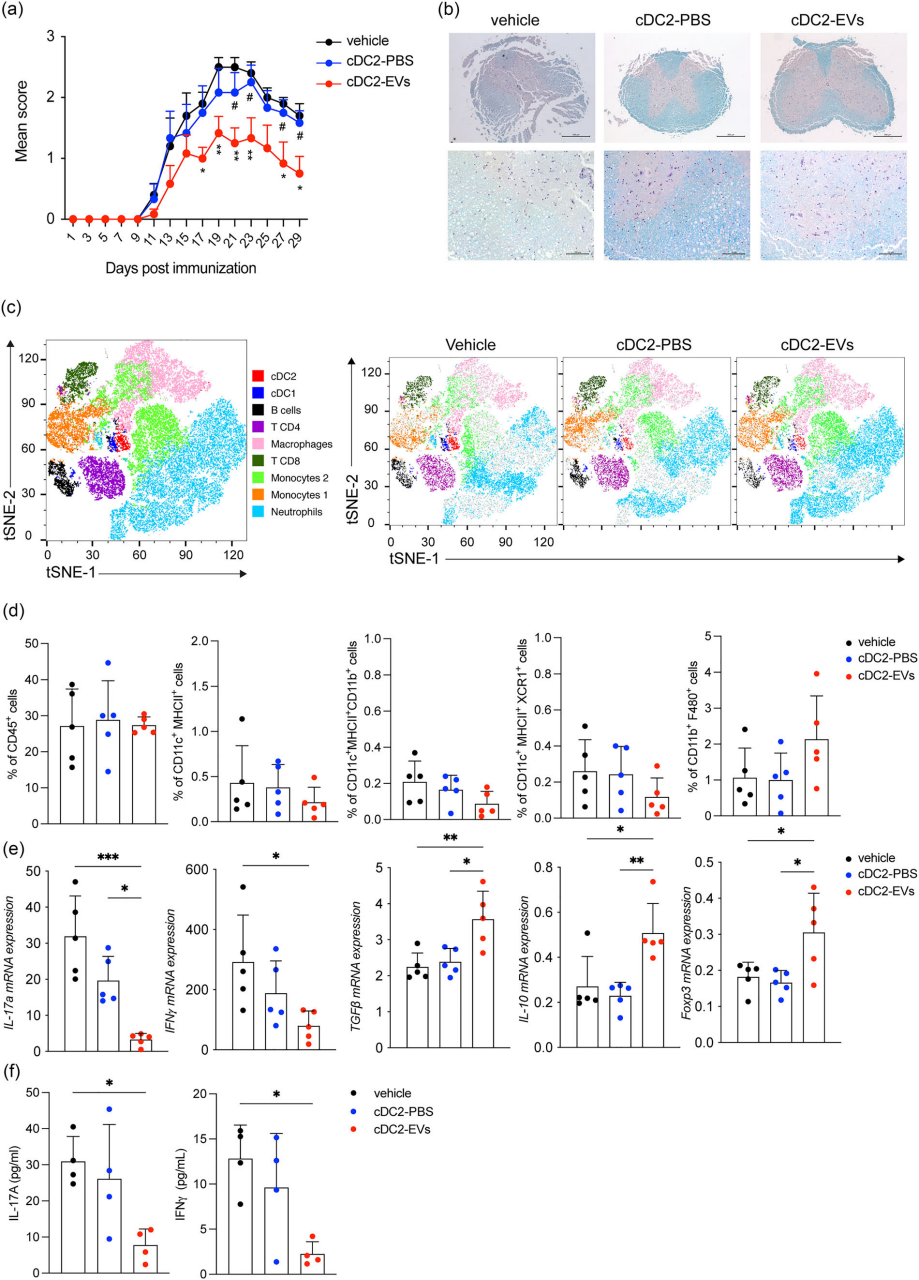
HAFSC‐EVs conditioned cDC2 induce immune‐regulatory functions in EAE. (a) EAE score in mice i.v injected with vehicle, cDC2 pretreated with vehicle (PBS) or HAFSC‐EVs^low^. Data are mean of daily EAE scores ± SD, *n* = 2, with *n* = 7 mice per group. *p < 0.05, **p < 0.01 (vehicle vs. cDC2‐EV), ^#^ *p* < 0.05 (cDC2‐PBS vs. cDC2‐EV) by two‐way ANOVA followed by Tukey recommended multiple comparison test. (b) Spinal cords were collected at sacrifice (30 days from immunization) from three mice per group, sectioned and stained with luxol fast blue for myelin. Scale Bar 500 and 100 μM. (c) tSNE plots of immune cells analysed in spinal cords of mice with EAE treated with vehicle, cDC2‐PBS and cDC2‐EVs showing nine clusters, coloured by density clustering and annotated by cell‐type identity. tSNE analysis was performed by FlowJo software. (d) Statistical analysis of immune infiltrate in spinal cords by flow cytometry. Data are reported as mean ± SD of the frequency of CD45^+^ immune cells. (e) Gene expression of IL‐17a, IFN‐𝛾, TGFβ, IL‐10, and Foxp3 in cervical lymph nodes activated in vitro with MOG peptide for 24 h and detected by Real Time PCR. mRNA for various cytokine transcripts were normalized by ß‐ACTIN expression. Fold change is relative to nonactivated lymph nodes. Data are mean ± SD *p < 0.05, **p < 0.01, ***p < 0.001, one‐way ANOVA followed by Bonferroni multiple comparison test. (f) Quantification of proteins in supernatants of cervical lymph nodes activated as in E and detected by ELISA. Data are means ± SD *p < 0.05, by one‐way ANOVA followed by Bonferroni multiple comparison test.

To assess the tolerogenic potential of HAFSC‐EV‐cDC2 in an in vivo setting, we performed experiments using a mouse model of EAE. Specifically, MOG_35‐55_ peptide‐pulsed cDC2, treated overnight with either HAFSC‐EVs (cDC2‐EVs) or vehicle PBS (cDC2‐PBS), were injected intravenously into WT mice with EAE, and progression of clinical disease was monitored over time as shown in Figure S4(A). Remarkably, mice receiving cDC2‐EVs showed a significant decrease in clinical disease compared with mice treated with cDC2‐PBS or vehicle (Figure 6a). Histological analysis of spinal cord tissues revealed improved myelin integrity (Figure 6b) and reduced immune cell infiltration (Figure 4b) in mice treated with cDC2‐EVs compared to the groups that received cDC2‐PBS or vehicle.

To gain insights into the immune‐mediated control exerted by cDC2‐EVs, we characterized the composition of infiltrating immune cells in the central nervous system (CNS). By performing flow cytometry analysis of multiple immune cell markers, we identified nine distinct cell clusters in the spinal cords of all experimental groups using t‐SNE analysis. These annotated clusters included neutrophils, two monocytes clusters, two T cell clusters (CD4^+^ and CD8^+^), macrophages, B cells and DCs (cDC1 and cDC2). Notably, tSNE analysis revealed differential cluster enrichment among the three experimental groups (vehicle, cDC2‐PBS and cDC2‐EVs) (Figure 6c). To understand which immune cell populations (i.e., CD45^+^) underwent the most significant changes, we quantified the percentage of immune cells (Figures 6d and 4c). While the differences were not statistically significant, there was a trend toward a decreased DCs infiltration (CD11c^+^MHCII^+^, CD11c^+^MHCII^+^CD11b^+^and CD11c^+^MHCII^+^XCR1^+^) and increased macrophage (CD11b^+^F480^+^) populations in mice treated with cDC2‐EVs compared to cDC2‐PBS (Figure 6d).

To evaluate the effects of cDC2‐EVs administration on T‐cell responses in vivo in the EAE model, we then isolated draining cervical lymph nodes from the different groups of mice and performed in vitro restimulation with the MOG peptide for 24 h. Our results showed that treatment with cDC2‐EVs significantly decreased the proportion of CD4^+^ expressing the cytokine transcripts for IL‐17 or IFN‐γ compared with mice receiving cDC2‐PBS or vehicle (Figure 6e). In addition, mice receiving cDC2‐EVs showed increased levels of CD4^+^ T cells expressing the immunoregulatory cytokines TGFß and IL‐10 and the regulatory marker Foxp3 compared with EAE mice treated with cDC2‐PBS or vehicle (Figure 6e). Moreover, in vitro restimulation of draining lymph nodes of mice treated with cDC2‐EVs resulted in lower levels of the pathogenic cytokines IL‐17 and IFN‐γ compared with mice treated with vehicle or cDC2‐PBS (Figure 6f).

Thus, these results demonstrate that HAFSC‐EV treated cDC2 are able to polarize T cells toward a regulatory phenotype. Moreover, these results support the notion that HAFSC‐EV treated cDC2 may be a promising therapeutic strategy to restore immune homeostasis in autoimmune diseases, particularly EAE.

## 4 DISCUSSION

In this study, we developed a system to educate inflammatory cDC2 to tolerogenic phenotype using EVs from HAFSCs. These EVs contain a repertoire of factors involved in immune cell reprogramming. We provided evidence that HAFSC‐EVs were preferentially taken up by the cDC2 compared to other immune cells, highlighting their specific targeting potential. Through our experiments we provide evidence that cDC2 conditioned with HAFSC–EVs acquired two key properties: the ability to secrete anti‐inflammatory cytokines and the capability to promote the differentiation of CD4^+^T cell into regulatory T cells. These findings highlight the immunomodulatory potential of HAFSC‐EVs and their role in reshaping immune functions.

Due to potential advantages like biocompatibility, biodegradation and efficient immune activation, EVs have gained attraction for the development of immune related disorders treatment. Notably, we show that in vivo transfer of autologous peptide‐pulsed HAFSC‐EV‐conditioned cDC2 conferred protective effects in a preclinical model of MS, that consisted in a significant reduction of pathogenic cytokines, decreased immune cells infiltration in the brain and spinal cord, and preservation of CNS tissue integrity.

EVs can interact with specific immune cells, including antigen‐presenting cells (e.g., DCs and other mononuclear phagocytes), to elicit immune responses or affect tissue and cell homeostasis or disease. Conventional DC subsets, cDC1 and cDC2, are associated with specialized functions (Guilliams et al., 2014) that can be strongly modulated by various environmental conditions. Dendritic cells are essential regulators of innate and adaptive immune responses as well as crucial cells in the maintenance of immune homeostasis. Recent studies have shown that EVs from various sources, particularly mesenchymal stem cells, have the potential to alter DC fate, leading to the activation of specific regulatory functions (Reis et al., 2018). Indeed, EVs transport almost all types of bioactive molecules (lipids, proteins, DNA, mRNAs, microRNAs and metabolites) that can act on recipient cells and alter their biological behaviour. Treatment of human DCs with MSC‐EVs arrested DC maturation, resulting in decreased expression of maturation and activation markers, decreased secretion of proinflammatory cytokines, including IL‐6 and IL‐12p70, and increased production of the anti‐inflammatory cytokine TGF‐β.

Our study is consistent with these reports and shows that HAFSC‐EVs activate regulatory functions in cDC subsets both in vitro and in vivo. They also add that amniotic EVs can act on mature cDCs, resulting in a phenotype similar to the recently described mregDCs (Maier et al., 2020). Indeed, HAFSC‐EVs suppressed LPS‐triggered cDC2 cytokine production and promoted regulatory T cell functions. Analysing the proteomic cargoes of HAFSC‐EVs, we found selective enrichment of some protein targets potentially involved in immune regulation. Notably, Muc 1 has been shown to support alternative activation of decidual macrophages, to limit proliferation of decidual regulatory CD56^+^ natural killer (NK) cells, and to downregulate their cytotoxic potential, including expression of cytotoxic mediator proteins (Redzovic et al., 2013). Moreover, GSK3 was enriched in HAFSC‐EVs. It is noteworthy that its role in modulating immune responses has only recently become known (Patel & Werstuck, 2021). Thus, it is possible that EVs deliver pools of regulatory proteins and genes to inflammatory cDC2 regulating their inflammatory activity and leading to restoration of tolerogenic function. Additionally, similarly to many EVs isolated and used in various studies, our EV preparation may co‐precipitate or bind other soluble factors. However, we cannot classify these factors as contaminants, as they may be biologically significant and closely associated with EVs. Notably, as outlined in the MISEV2023 guidelines (Welsh et al., 2024), some molecules that co‐isolate with EVs, such as proteins, nucleic acids, sugars, and lipids, may be part of a dynamic EV ‘corona’. Recently, it has been shown that the so‐called ‘EV corona’ can greatly affect the behaviour of synthesized particulates in vivo, including cellular targeting (Heidarzadeh et al., 2023; Liam‐Or et al., 2024). The protein corona is formed when EVs come into contact with biological fluids, and proteins are adsorbed onto the EV surface. For functional studies, MISEV2023 guidelines (Welsh et al., 2024) outline that recommend using selected EV negative controls to assess the contribution of ‘background’ EV activity, such as EVs present in culture medium components. For cell culture‐derived EVs, this might mean an unconditioned medium that has been processed in the same way as conditioned medium (i.e., to separate any EVs that may be present in culture medium components). Related to this point, in this study we had the opportunity to confirm that the unconditioned medium, processed similarly to that of the conditioned medium by HAFSCs, did not yield measurable EV counts. Consequently, these data suggest that the functional effects on cDC2 and their resulting co‐culture in vitro or in vivo effects on EAE are mediated by EVs from HAFSC and not from other molecules. In addition, our data suggest that the functional effects of HAFSC‐EVs on cDC2 may also involve an EV composition “corona” that could potentially influence cDC2 functions.

Understanding the cell specificity of EVs interaction with DC populations and uptake is critical to facilitate the development of EVs as valuable diagnostics and therapeutics. Intriguingly, our results show that EV uptake can be mediated in part by the integrin CD29. However, it is noteworthy that not all vesicles were dependent on CD29 and accounted for approximately 40–50% of total uptake. This suggests that other integrins or alternative mechanisms may be involved in the uptake of this particular type of vesicle. Further studies are needed to explore these additional pathways involved in EVs uptake.

Previous studies have highlighted the widespread uptake of EVs by different cell types, suggesting that EVs are internalized through multiple pathways (Mathieu et al., 2019; Mulcahy et al., 2014; Svensson et al., 2013). Further results suggest that EV uptake may be a selective process in which certain recipient cells are able to internalize specific EVs compared to others (Rana et al., 2012). According to these data, our study provides new insights by demonstrating for the first time that selectively activated cDC2 cells show a greater propensity to take up cargo from HAFSC‐EVs compared with their cDC1 counterparts. This observation is of great relevance, especially in microenvironments where both cDC subsets coexist, which could lead to the acquisition of different effector functions.

Integrin beta‐1 (ITGB1), also known as CD29, is a cell surface receptor that in humans is encoded by the ITGB1 gene (Goodfellow et al., 1989). Integrin β1, which is one of the most common subunits in the integrin family is widely known for its role in facilitating cell‐to‐cell and cell‐to‐matrix interactions (Bax et al., 2003). Lipid composition and the presence of tetraspanin proteins on EVs membranes regulated by donor cells are partly responsible for the tropism of EVs toward recipient cells (Rana et al., 2012). Interestingly, our results show that the β1 integrin CD29 was abundantly expressed on the surface of cDC2. Although it remains unclear whether the HAFSC‐EVs, used in this study, have the same target selectivity for human cDCs in vivo, our data may suggest that reprogramming of inflammatory cDC2 by HAFSC‐EVs could be a possible mechanism to explain the lower frequency of MS relapses observed during pregnancy. Of note, the most significant decrease in relapses occurs during the third trimester in which HAFSCs were obtained, followed by disease activity and relapse rates after delivery (Confavreux et al., 1998). Of note, the HAFSC lines used to produce EVs in this study were derived from stem cells obtained from amniocentesis during the third trimester of pregnancy. This information adds an interesting context to our findings, as it suggests a potential link between the source of the EVs (HAFSCs from the third trimester) and the observed reduction in MS relapses during the same period.

Several reports demonstrate the immunosuppressive properties of amniotic cells, which can be attributed to their role in maintaining feto‐maternal tolerance during pregnancy (Luo et al., 2014; Mezzasoma et al., 2022). Small EVs secreted by human amniotic fluid stromal cells have been observed to contain specific cargo proteins and nucleic acids that reflect various protective and immunoregulatory functions of parental cells (Ohara et al., 2018; Romani et al., 2015; Sedrakyan et al., 2017; Takov et al., 2020). Conventional DC2 reprogramming by HAFSC‐EVs may be an additional mechanism by which HAFSCs may be able to regulate immune functions and suppress T lymphocyte proliferation when co‐cultured with human and mouse PBMCs (Kang et al., 2012; Magatti et al., 2008; Roelen et al., 2009; Romani et al., 2015). In addition, DC co‐cultured with HAFSC‐EVs, may also acquire the ability to release additional regulatory EVs further enhancing immune regulatory responses.

Microglial cells in the brain, although abundant and strategically located at the CNS‐immune interface, are unable to process and present myelin antigens to naïve T cells. On the other hand, CNS‐associated cDCs, particularly the cDC2 subset, have been identified as highly professional antigen‐presenting cells (APCs) and as the only cellular bridging element that can facilitate effective T cell‐CNS interactions (Mundt et al., 2019). Our results show that administration of cDC2 conditioned with HAFSC‐EVs leads to an improvement in EAE and an enhanced Treg response. These results strongly suggest that the protective effects observed in EAE are due to reprogramming of mature cDC2 by EVs, which not only induce secretion of anti‐inflammatory cytokines but also release additional EVs that can reach the CNS. This dual mechanism of action likely contributes to the restoration of immune homeostasis and reversal of neuronal degeneration and autoimmune reaction. There are some limitations to this study. While our study focused on the effects of HAFSC‐EVs on cDC2, further studies are needed to elucidate the specific molecular mechanisms by which HAFSC‐EVs reprogram cDC2 function. This may impact the translation of these findings into therapeutic applications. Unravelling the cargo and signalling pathways involved in EV‐mediated reprogramming could provide valuable insights into the immunomodulatory properties of EVs and their potential therapeutic applications also in combination therapies, or investigating the long‐term effects of EV treatment. In addition, it is important to consider the potential interactions and crosstalk between EVs and other immune cell populations. Investigating the effects of HAFSC‐EVs on other immune cells, such as T cells, B cells, and macrophages, could provide a more comprehensive understanding of the overall immune response and potential. In particular, they may affect the potential collaboration or crosstalk with T cells, B cells and macrophages and these interactions may influence the overall immune response. In addition, further studies are needed to explore the in vivo mechanisms by which the transfer of EV‐treated cDC2 cells leads to improvement in mice with EAE. This will be critical for translating these results into the therapeutic potential of HAFSC‐EVs and HAFSC‐EVs reprogrammed cDC2.

In conclusion, our in vitro study demonstrates that HAFSC‐derived EVs are selectively taken up by the cDC2 subset, leading to the development of a tolerogenic DC population that releases anti‐inflammatory molecules that in turn promote protection against EAE. Our results highlight the potential of EV‐mediated reprogramming as a means of active and effective immune regulation in both the periphery and central nervous system in the context of autoimmunity. These results are promising for the development of novel therapeutic strategies targeting pathogenic cDC2 cells and restoring immune homeostasis in autoimmune diseases. However, further studies are needed to fully understand the mechanisms of EV‐mediated reprogramming and to evaluate its translational potential in the clinical setting.

## AUTHOR CONTRIBUTIONS

**Giorgia Manni**: Conceptualization (equal); investigation (lead); writing—original draft (equal); writing—review and editing (equal). **Marco Gargaro**: Investigation (equal). **Doriana Ricciuti**: Investigation (equal). **Simona Fontana**: Methodology (equal). **Eleonora Padiglioni**: Investigation (equal). **Marco Cipolloni**: Investigation (equal). **Tommaso Mazza**: Methodology (equal). **Jessica Rosati**: Investigation (equal). **Alessandra Di Veroli**: Investigation (equal). **Giulia Mencarelli**: Investigation (equal). **Benedetta Pieroni**: Investigation (equal). **Giulia Scalisi**: Investigation (equal). **Francesco Sarnari**: Investigation (equal). **Alessandro Di Michele**: Investigation (equal). **Luisa Pascucci**: Investigation (equal). **Francesca De Franco**: Investigation (equal). **Teresa Zelante**: Investigation (equal). **Cinzia Antognelli**: Resources (equal). **Gabriele Cruciani**: Resources (equal). **Vincenzo Nicola Talesa**: Resources (equal). **Rita Romani**: Conceptualization (equal); investigation (equal); resources (equal). **Francesca Fallarino**: Conceptualization (equal); resources (equal); visualization (equal); writing—original draft (equal); writing—review and editing (equal).

## CONFLICT OF INTEREST STATEMENT

The authors declare no competing interests.

## Supporting information

Supporting Information

Supporting Information

Supporting Information

Supporting Information

## Data Availability

All data are available in the main text and/or the Supplementary Materials, tables and video.
