# Supplementary material for: Amniotic fluid stem cell‐derived extracellular vesicles educate type 2 conventional dendritic cells to rescue autoimmune disorders in a multiple sclerosis mouse model
Source: J Extracell Vesicles. 2024 Jun 6;13(6):e12446. doi: 10.1002/jev2.12446 (PMC11156524; doi:10.1002/jev2.12446)
Supplement: Supplementary file 1 — Supporting Information [file JEV2-13-e12446-s001.docx]

Supplementary Materials for

***Amniotic fluid stem cell derived extracellular vesicles educate type 2 conventional dendritic cells to rescue autoimmune disorders.***

Giorgia Manni *et al.*

*Corresponding author. Email: [francesca.fallarino@unipg.it](mailto:francesca.fallarino@unipg.it)

Supplementary Materials and Methods

**Flow cytometry**

Phenotypical characterization of HAFSCs was performed by flow cytometry analysis. Briefly, cells were culture with or without serum for 24h. After that cells were harvested by trypsinization, washed with phosphate-buffered saline supplemented with FBS (3%) and stained with antibodies reacting to the following antigens: HLA A, B, C (Beckton Dickinson), CD105 (Immunotools), CD90, Oct4 and Kfl4 (BioLegend). Cells were acquired on an LSR Fortessa (BD Biosciences) flow cytometer.

Flow cytometry analysis was also used to evaluate HAFSC-EVs uptake to recipient cells in total splenocytes (as described in materials and methods) and HAFSC-EVs biodistribution in *ex-vivo* experiments. Mice i.v. injected with vesicles were sacrificed at 6 and 24 hours after injection. Blood, spleen, lymph nodes, lung, liver and brain were isolated from sacrified mice and organs were processed for FACS analysis. Cell suspensions from brain, lung and liver were stained with CD45 monoclonal antibody (BioLegend) to reveal immune components. Blood, spleen and lymph nodes were stained with CD45, CD4, CD8, CD11c, CD11b, B220, F480 antibodies (BioLegend) to reveal the different uptake levels of HAFSC-EVs to recipient immune cells.

**HAFSC-EVs staining**

In order to study the uptake and internalization of EVs in recipient cells, vesicles were labeled with Dil dye (1,1'-Dioctadecyl-3,3,3',3'-Tetramethylindocarbocyanine Perchlorate ('DiI'; DiIC_18_(3)) (Thermo Fisher Scientific, Waltham, MA, USA). Briefly, Dil dye was added to 30 ml of conditioned medium at a concentration of 10 μM and incubated for 20 minutes at 37°C in the dark. Extracellular vesicles (EVs) were then isolated by the differential ultracentrifugation method as described in the extracellular vesicle isolation sections of the Methodology. After ultracentrifugation, the purified EVs were washed twice with sterile and filtered PBS. The pellet obtained was then resuspended in sterile and filtered PBS for subsequent studies.

**Transmission electron microscopy**

Twenty μl of fresh vesicle suspension was placed on Parafilm. Formvar coated copper grids were gently deposited on the drops with the coated side towards the suspension. After 1-hr incubation, grids were put on a drop of PBS and floated 5 minutes with the coated side facing the buffer. The grids were washed in distilled water and then transferred for 5 minutes on a drop of 2% uranyl-acetate dissolved in distilled water. After washing in distilled water, the grids were air dried and observed under a Philips EM 208 transmission electron microscope equipped with a digital camera (Center for Electron and Fluorescent Microscopy (CUMEF) - University of Perugia).

**Differential expression analysis**

To better understand whether the *Itgb1* and *Itgb3* genes appear differentially expressed in the comparison between cDC1 and cDC2 cell populations, we used our previously published dataset with GEO accession number GSE203450. In brief, microarray data of untreated cDC1 and cDC2 sort-purified cells from the bone marrow of healthy C57BL/6 mice were selected and normalized for a differential gene expression analysis using limma-voom package (Ritchie et al., 2015). Differentially expressed genes were represented in a volcano plot using the ggplot package. All the data processing was carried out in the R environment.

**Supplementary Figures**

**Fig. S1.**


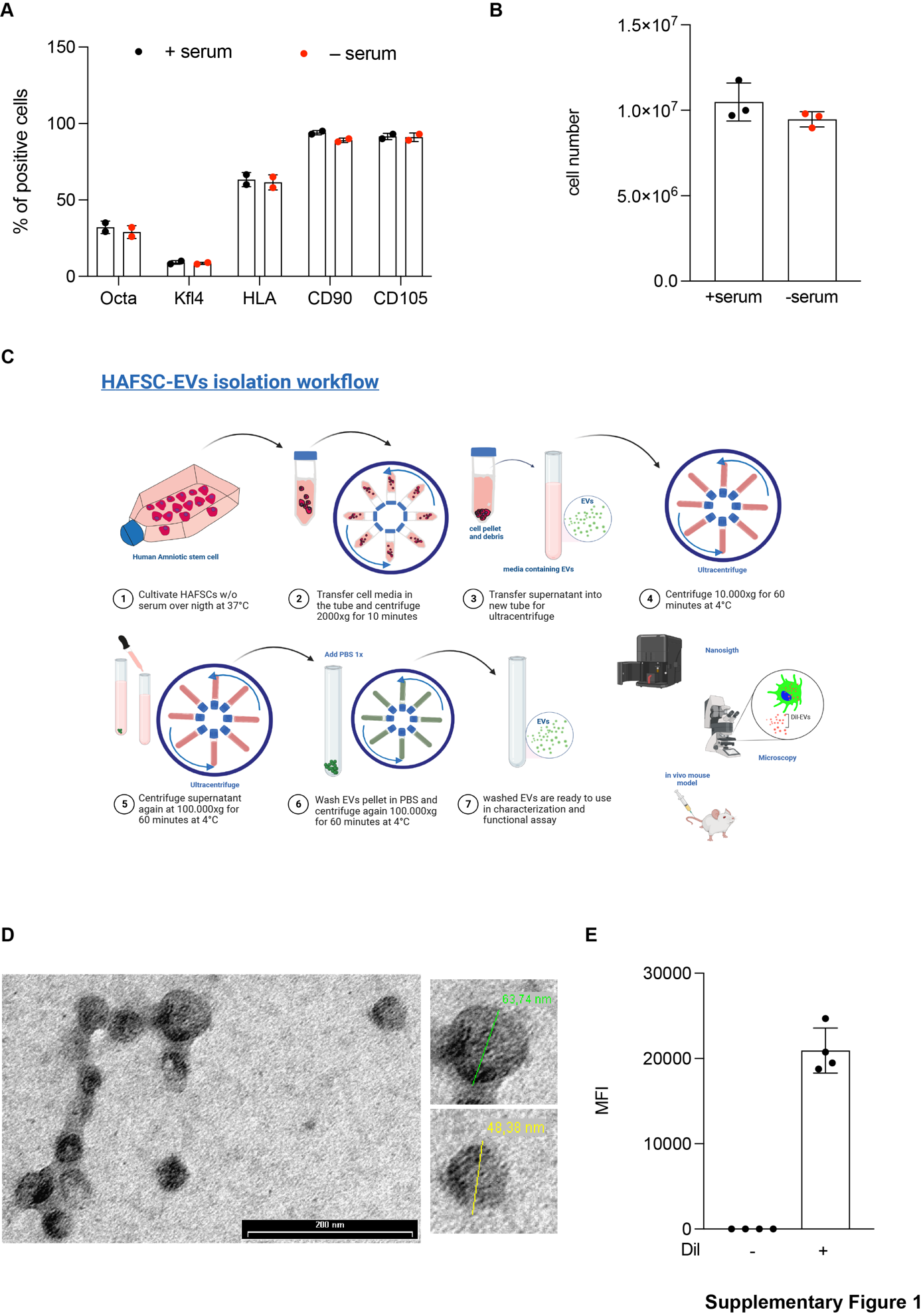


**Fig. S1: HAFSC secrete EVs in their conditionated medium. (A)** Analysis of stromal markers expression **(**Oct4, Kfl4, HLA, CD105 and CD90) by flow cytometry in amniotic fluid stem cells cultured 24h in media with or without serum. Data are mean ± S.D. of two independent experiments, each performed in triplicate. **(B)** Amniotic fluid stem cells count after 24h culture in media with or without serum. Data are mean ± S.D. of three independent experiments. **(C)** Schematic illustration of HAFSC-EVs isolation. 1: cultivate HAFSC w/o serum over night at 37°C; 2: transfer cell media in the tube and centrifuge 2000xg for 10 minutes; 3: transfer supernatant into new tube for ultracentrifuge; 4: centrifuge 10000xg for 60 minutes at 4°C; 5: centrifuge supernatant again at 100000xg for 60 minutes at 4°C; 6: wash EVs pellet in PBS and centrifuge again at 100000xg for 60 minutes at 4°C; 7: washed EVs are ready to use in characterization and functional assay. **(D)** HAFSC-EVs morphology and diameter measure observed by TEM. **(E)** EV-Dil staining analyzed by flow cytometry. Data are mean ± S.D. of four independent experiments.

Fig. S2.


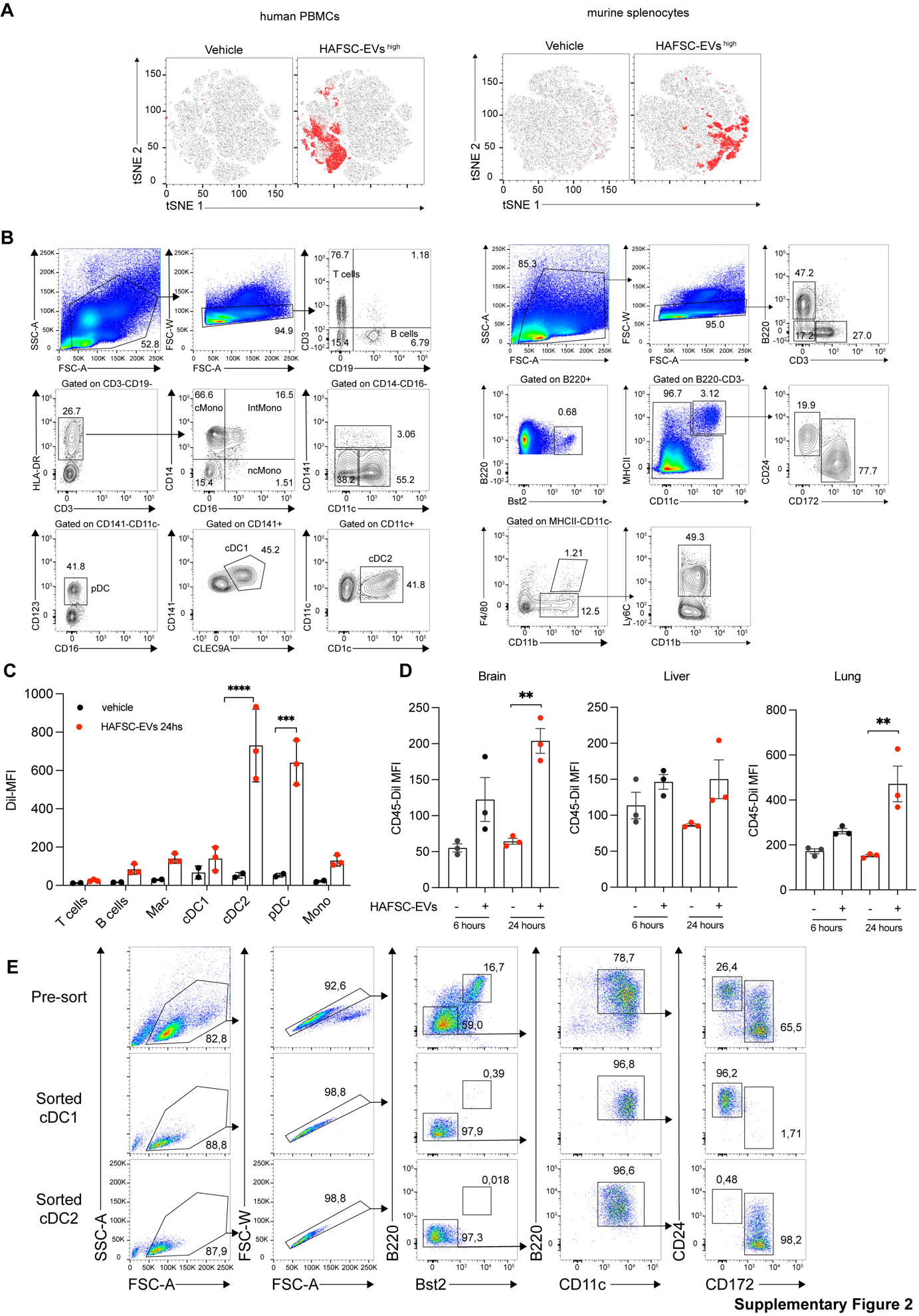


**Supplementary Figure 2: HAFSC-EVs uptake in vitro and in vivo. (A)** 2-dimensional t-SNE analysis (FlowJo) represents the several cell populations before and after treatment with HAFSC-EVs and the distribution of EVs (red) on total human PBMCs and murine splenocytes (grey). **(B)** Flow cytometry gating strategy used to identify specific immune cell populations in human PBMCs and murine splenocytes. **(C)** HAFSC-EVs *in vivo* uptake. Dil-HAFSC-EV or vehicle were injected intravenously into mice, and spleens were harvested after 24 hours of treatment. Dil signal was evaluated ex-vivo by flow cytometry in different immune cell populations. Data are represented as mean ± SD of MFI (mean fluorescent intensity) of three independent experiments (*** p < 0.001, **** p < 0.0001 by two-way ANOVA with Bonferroni’s multiple comparison test). **(D)** Dil-HAFSC-EVs were injected i.v. as described in C and vesicles biodistribution was measured ex vivo by flow cytometry at 6- and 24-hours post-injection in CD45^+^ cells of different organs (brain, lung and liver). Appreciable signal was detected in CD45^+^ cells of brain and lung both at 6 and 24 hours. Data are represented as MFI ± SD (n=3, *p< 0.05 by one-way ANOVA with Bonferroni’s multiple comparison test). **(E)** Flow cytometry gating strategy to identify purity of bone marrow derived dendritic cells used to evaluate HAFSC-EVs in in vitro experiments.

Fig. S3.


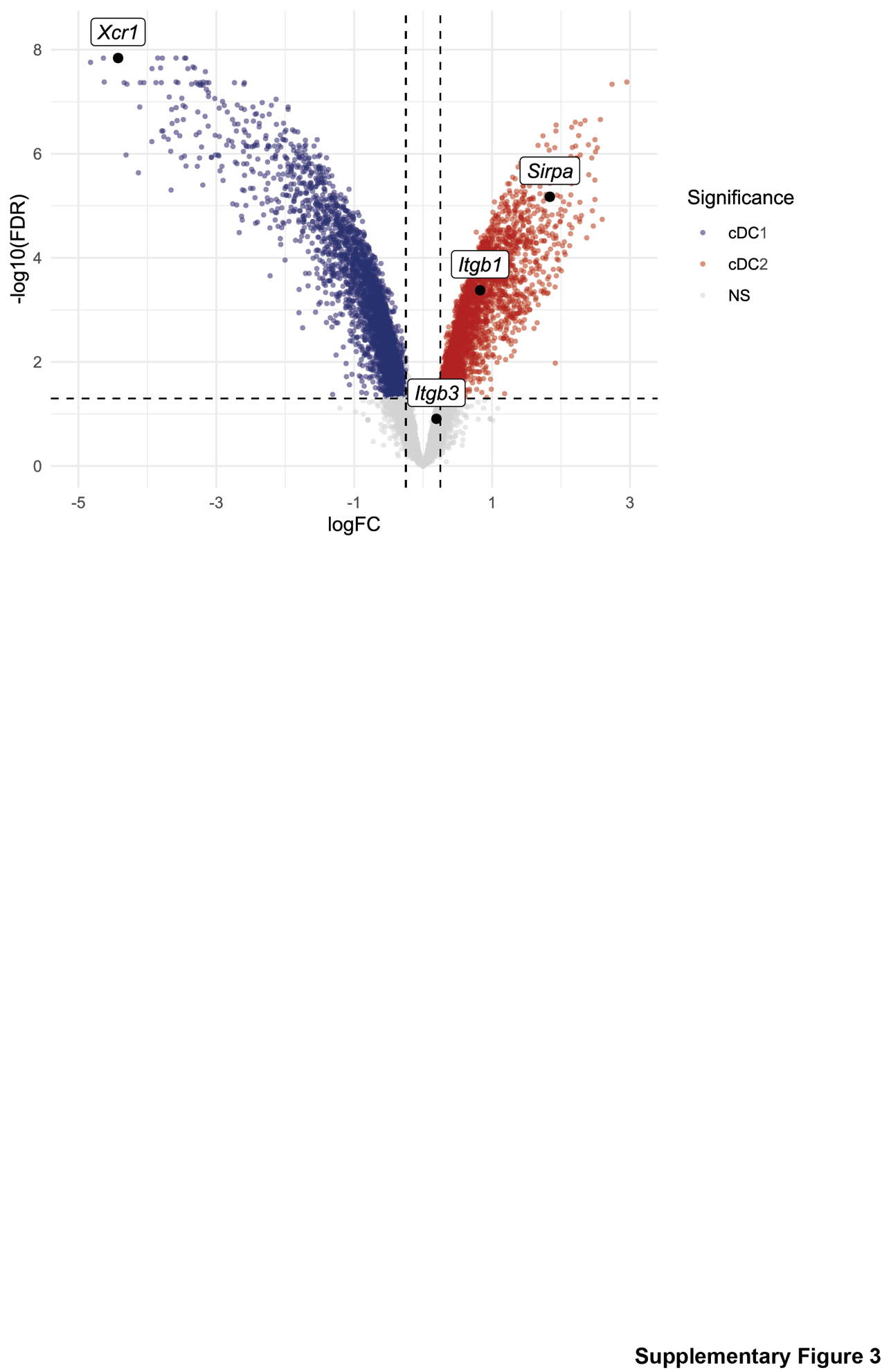


**Supplementary Figure 3: Differential gene expression in bone marrow-derived cDC1 compared to cDC2 cells.** Volcano plot depicting differentially expressed genes in cDC1 and cDC2 cells. Red dots represent genes expressed at higher levels in cDC2 cells (e.g. *Sirpa*) while blue dots represent genes with higher expression levels in cDC1 cells (e.g. *Xcr1*). The figure highlights *Itgb1* and *Itgb3* genes. Y-axis denotes − log10 P values (FDR) while X-axis depicts log2 fold change (logFC) values. Differential gene expression was calculated using Limma-Voom, and the volcano plot was generated using the ggplot package with logFC cut-off of 0.25 and FDR cut-off of 0.05.

**Fig. S4.**

**
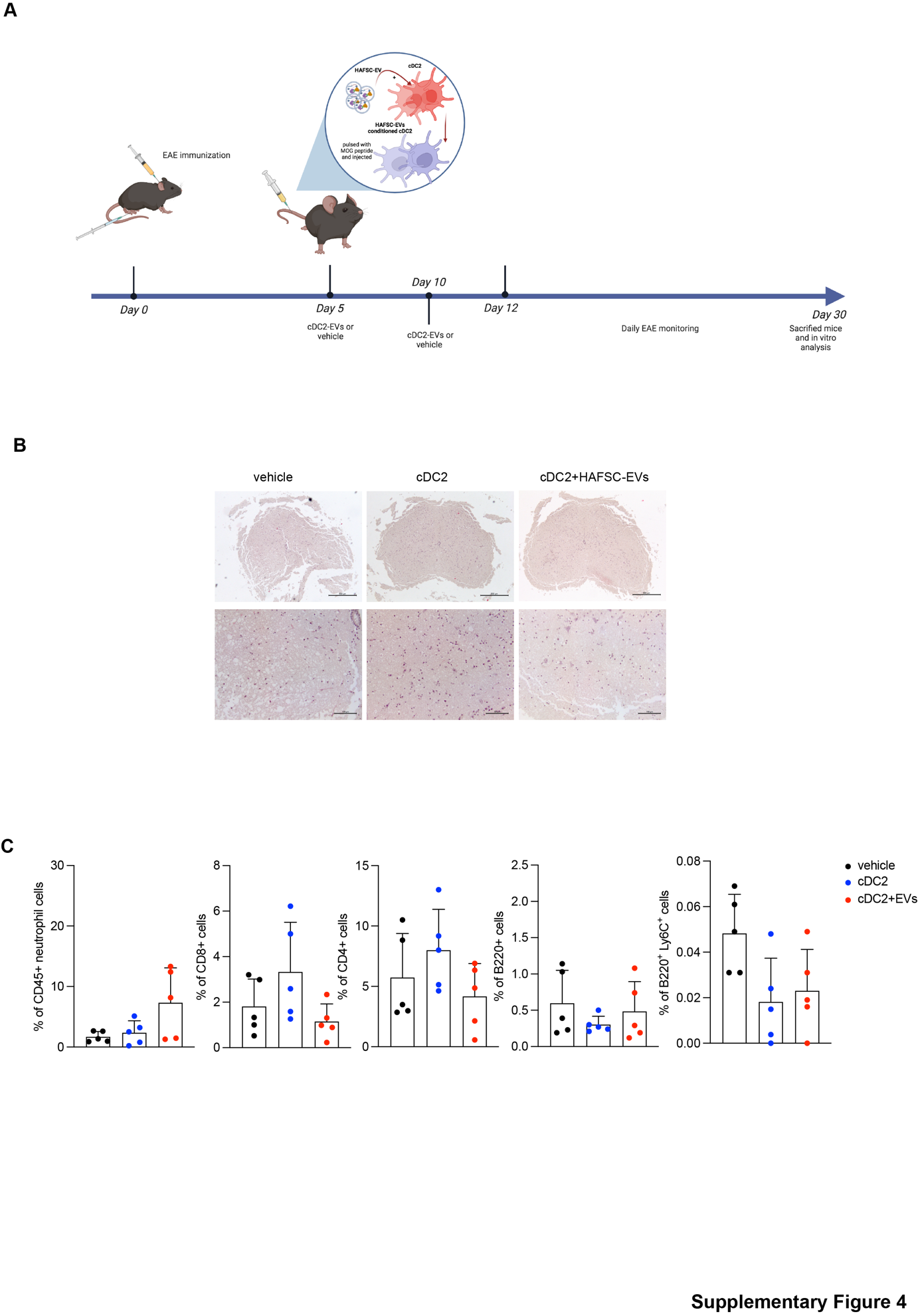
**

**Supplementary Figure 4: HAFSC-EVs conditioned cDC2 protect mice with EAE. (A)** Mice were immunized with MOG peptide on day 0 and given pertussis toxin on day 2. cDC2, treated or not with HAFSC-EVs, were injected at day 5 and 10 post immunization. Onset of clinical illness is observed on days 10-12, with peak severity at days 12-18. Mice were scored daily and sacrificed at day 30. **(B)** H&E staining of spinal cord sections of mice treated as in A to visualize immune infiltrate. Scale Bar 500 μM and 100 μM. Images were representative of one mouse of three analyzed. **(C)** Percentage of immune infiltrate cells in spinal cord by flow cytometer. Data are reported as mean ± SD of the frequency of CD45^+^ immune cells.

Data S1. (separate file)

Table 2. HAFSC-EVs proteins

Table S1. HAFSC-EVs miRNAs

Movie S1.

Dil-HAFSC-EVs internalization into cDC1.

Movie S2.

Dil-HAFSC-EVs internalization into cDC2.

**References**

Ritchie, M.E., Phipson, B., Wu, D., Hu, Y., Law, C.W., Shi, W., and Smyth, G.K. (2015). limma powers differential expression analyses for RNA-sequencing and microarray studies. Nucleic Acids Res *43*, e47.
